# Assessing oceans’ health through biopsy sampling in free-ranging marine mammals: a systematic review (2014–2024)

**DOI:** 10.1007/s11356-026-38163-3

**Published:** 2026-09-01

**Authors:** Suelen Goulart, Giulia Galani, Lucas Fazardo de Lima, Isadora Nicole Lara Piccinin, Felipe da Silva Valente, Aline Nunes, Eduardo Pires Renault-Braga, Marcelo Maraschin

**Affiliations:** 1 Laboratório de Metabolômica E Bioquímica Aplicada, Centro de Ciências Biológicas, Universidade Federal de Santa Catarina, Florianópolis, Brazil; 2 Instituto de Biociências, Universidade Estadual Paulista - UNESP, Botucatu, São Paulo Brazil; 3 Instituto Australis, Centro Nacional de Conservação da Baleia Franca, Imbituba, Brazil

**Keywords:** POPs, Non-invasive sampling, Pollution monitoring, Inorganic contaminants, Phthalates, Emerging pollutants, Cetaceans, Pinnipeds

## Abstract

Marine mammals are ecological sentinels of ocean health. As apex predators, they can bioaccumulate contaminants through aquatic food webs. In this context, this systematic review evaluates the role of minimally invasive biopsy sampling in assessing contaminant exposure across free-ranging populations. Following PRISMA guidelines, 31 studies were evaluated, covering the period from 2014 to 2024, and sourced from the CAPES Periodicals Portal database. Results indicate a primary focus on persistent organic pollutants (POPs; 74.19%), such as PCBs and DDTs, with a notable geographic bias toward cetaceans in the Northern Hemisphere (47.4% in the Americas). Cetaceans, especially bottlenose dolphins (*Tursiops truncatus*), were significantly overrepresented (93.94% of studies), while studies on the Carnivora order, focused on pinnipeds, were scarce (6.06%). Inorganic contaminants were underrepresented, despite concerning mercury levels in dolphins and chromium-induced genotoxicity in fin whales. Interestingly, emerging contaminants, like phthalates, were detected in only two studies. DEHP, a hazardous plasticizer, was detected in three cetacean species. Thus, the urgent need to expand monitoring efforts to underrepresented regions and to integrate biopsy analysis with other approaches (e.g., multi-omics, isotopic analyses), long-term research, and satellite telemetry to map pollution hotspots is emphasized. Standardizing protocols and establishing global biobanks are essential for data harmonization. This work highlights the critical role of biopsies within the One Health framework, connecting marine pollution data to human and ecosystem resilience.

**Graphical Abstract:**

**Figure:**
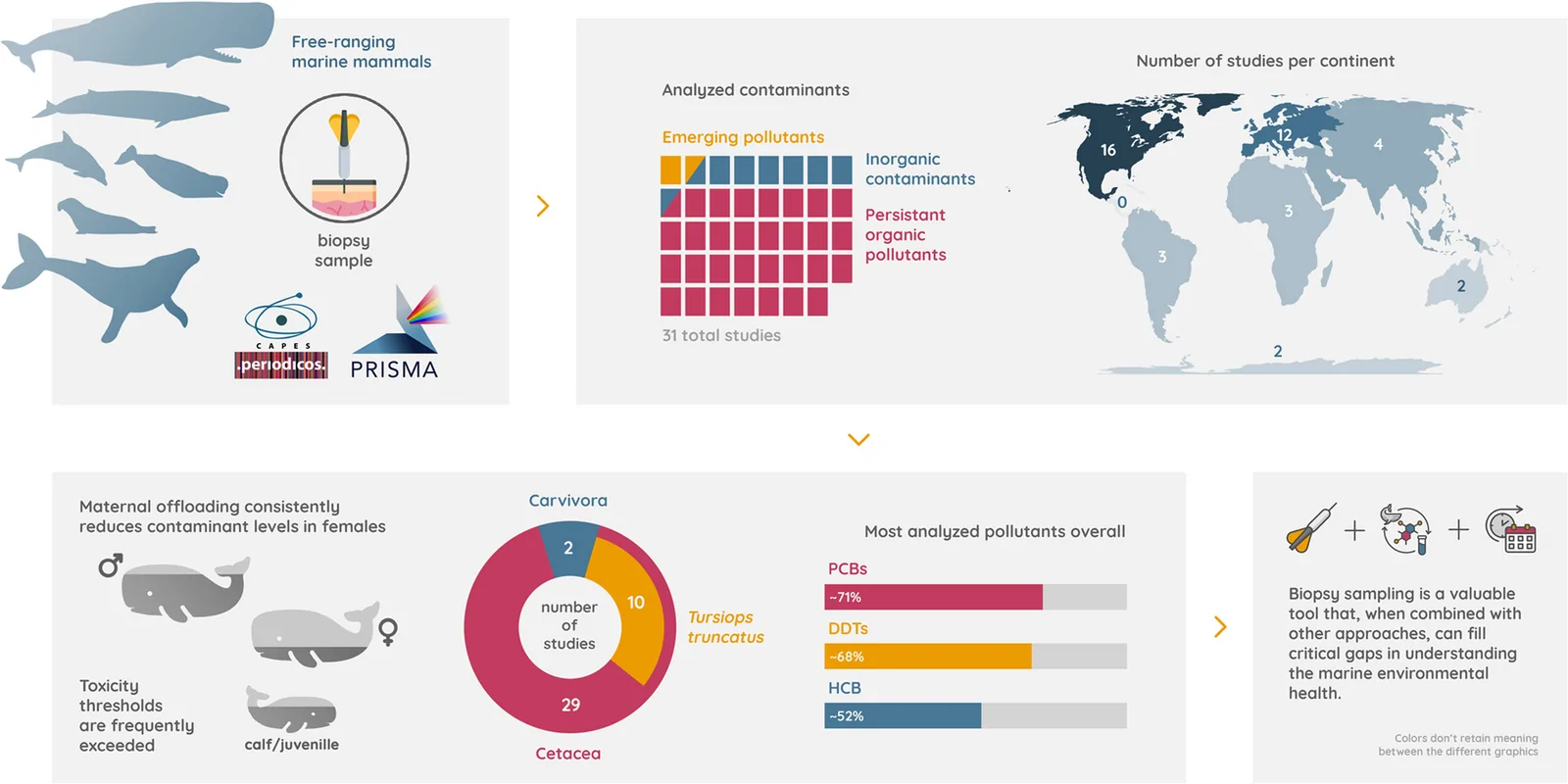

## Introduction

Marine ecosystems play a crucial role in maintaining global environmental balance through climate regulation, biogeochemical nutrient cycling, and biodiversity conservation (Dong et al. 2024). However, these ecosystems face increasing threats from anthropogenic impacts, including chemical pollution, plastic waste disposal, and climate change (MacLeod et al. 2021; Williamson and Guinder 2021; Williamson et al. (Williamson, et al., 2021); Schaap et al. 2023). In this context, continuous monitoring of the health of seas and oceans is essential for understanding the effects of anthropogenic pressures and developing effective mitigation and conservation strategies (Bowler et al. 2020) in a scenario of continuous challenges resulting from pollution and climate change.

Marine mammals stand out as environmental bioindicators due to their high trophic level and sensitivity to contaminants (Reckendorf et al. 2023). As apex predators, these animals accumulate pollutants through bioaccumulation and biomagnification processes, including potentially toxic metals, persistent organic pollutants (POPs), microplastics, and phthalates (Kruse et al. 2023; Reckendorf et al. 2023; Williams et al. 2023; Andvik et al. 2024). Thus, the analysis of contaminants in marine mammals reflects not only individual health but also the environmental quality of their ecosystems (Williams et al. 2023). However, assessing contamination levels in these groups presents significant methodological challenges, particularly due to reliance on post-mortem samples. Traditional methods, such as necropsies, can be useful in understanding how anthropogenic factors, including bycatch, vessel collisions, and pollution, affect marine mammals (Méndez-Fernandez et al. 2016; Schoeman et al. 2020; IJsseldijk et al. 2021). However, assessing contaminants in stranded animals can be challenging due to considerable limitations—including the opportunistic nature of samples, decomposition, under-representation of healthy populations, and reliance on debilitated or deceased individuals—resulting in biased outcomes that impede continuous and representative health monitoring of marine mammal populations (Reckendorf et al. 2023; IJsseldijk et al. 2024). Additionally, necropsy-based sampling poses a relevant constraint for the purpose of metabolomics analysis due to the near impossibility of effectively quenching cell metabolism. Given these limitations, the development of non-lethal techniques that enable accurate and minimally invasive assessment of contaminant exposure, such as skin biopsy, becomes imperative (Cáceres-Saez et al. 2021).

Biopsy sampling has emerged as a valuable tool not only for being non-lethal and minimally invasive but also for enabling the collection of tissue samples from live individuals without prolonged capture or highly stressful procedures (Sinclair et al. 2015). Unlike necropsies, which are limited to deceased animals, biopsies allow monitoring of active populations, thereby minimizing disturbance to the individuals under study (Funasaka et al. 2024). Even though the biopsy methodology requires precautions, such as the correct sampling dispositive, training of researchers to minimize the risk to animals and chances of mark-recapture (Noren and Mocklin 2012; Hunt et al. 2013; Booth et al. 2020), the technique reduces the risk of significant injury and physiological stress associated with extended handling, offering a viable alternative for obtaining representative data on contaminant exposure (Hunt et al. 2013; Funasaka et al. 2024). Importantly, biopsy sampling enables effective metabolic quenching, producing reliable samples for metabolomics studies. An additional advantage is the ability to integrate ecological data—such as movement patterns obtained via telemetry and dietary information inferred from isotopic analysis—thus enabling a more comprehensive assessment of organism health and environmental impacts (Troina et al. 2021; Kleivane et al. 2022).

Over the last few years, technological advancements have broadened the application of biopsies to detect contaminants, even in small samples. Analytical techniques, such as gas chromatography coupled with mass spectrometry (GC-MS), are essential for identifying organic pollutants in biological tissues (Beale et al. 2018; Schulz et al. 2023). Advances in molecular biology resulting from the development of PCR techniques have also enabled the detection of genetic and physiological responses in organisms exposed to environmental stressors, including contaminants (Panti et al. 2011; Fossi et al. 2014; Chakraborty et al. 2022). In the context of marine mammals, these tools enhance the precision of environmental studies and contribute to more effective health monitoring strategies, reinforcing the role of biopsies as a valuable approach in conservation and ecological assessments (Reckendorf et al. 2023).

In this context, this systematic review aimed to assess the applicability of biopsy sampling as a tool for detecting environmental contaminants in free-ranging marine mammals. Beyond its relevance for evaluating pollutant exposure, this review also explores the potential of integrating biopsy data with ecological information to improve environmental monitoring efforts and support the development of more effective conservation strategies.

## Material and methods

### Study design

This systematic review followed the PRISMA (Preferred Reporting Items for Systematic Reviews and Meta-Analyses) protocol to ensure transparency, reproducibility, and methodological rigor. The study aimed to investigate how biopsies from marine mammals can contribute to environmental contamination studies, highlighting the advantages and disadvantages of this experimental approach. The search was conducted considering the scientific information available in the CAPES Journals Portal database (Coordination of Improvement of Higher Education Personnel of the Ministry of Education in Brazil; https://www-periodicos-capes-gov-br.ez46.periodicos.capes.gov.br/; Nunes et al. 2025).

The search used the descriptors “biopsy” and “marine mammals” and was limited to a 10-year timeframe (2014–2024). The initial search yielded a comprehensive list of articles, which were subsequently screened and filtered according to predefined inclusion and exclusion criteria, as described further.

### Quality assessment

To ensure the reliability and objectivity of the study selection process, a rigorous quality assessment was implemented. The initial screening of articles was conducted independently by two researchers (S.G. and G.G.M.) on consecutive days to minimize bias and ensure technical reliability. On the third day, a third researcher (I.N.L.P.) reviewed the results of the two independent screenings, resolving any discrepancies and consolidating the final list of articles. The use of independent reviewers and a consensus-based decision-making process strengthened the validity of the study selection.

### Data extraction

The data extraction process was conducted systematically to ensure consistency and accuracy. The dataset was compiled and saved as a Microsoft Excel (version 365) datasheet. After applying the initial search filters, 255 articles were identified. These articles were compiled into a comprehensive table containing the following information: title, journal, publication year, and abstract. This table served as the basis for further analysis and screening of data. Subsequent filtering was performed to refine the selection based on more specific criteria (which will be presented next). Articles that did not meet the inclusion criteria were excluded, resulting in a final list of 31 articles. The extracted data were organized to facilitate the analysis of the relationship between biopsy techniques in marine mammals and their application in environmental contamination studies.

### Inclusion/exclusion criteria

The initial search in the CAPES Journals Portal database applied the following inclusion criteria: (1) original scientific articles, (2) peer-reviewed, (3) published in English, and (4) within the time frame of 2014–2024. From the initial pool of 255 articles, more detailed screening was conducted by applying additional exclusion criteria. First, 13 articles were excluded for being outside the specified time frame of interest. Additionally, four review articles, five non-article publications (e.g., editorials and book chapters), and one duplicate were also removed.

The remaining articles were further screened based on the following inclusion criteria: (1) studies that used biopsy as a sampling technique, (2) studies conducted on live marine mammals, and (3) studies focused on the analysis of environmental contaminants (e.g., POPs, inorganic and emerging contaminants) in biopsied marine mammals.

## Results and discussion

### Study selection and characteristics

**Fig. 1:**
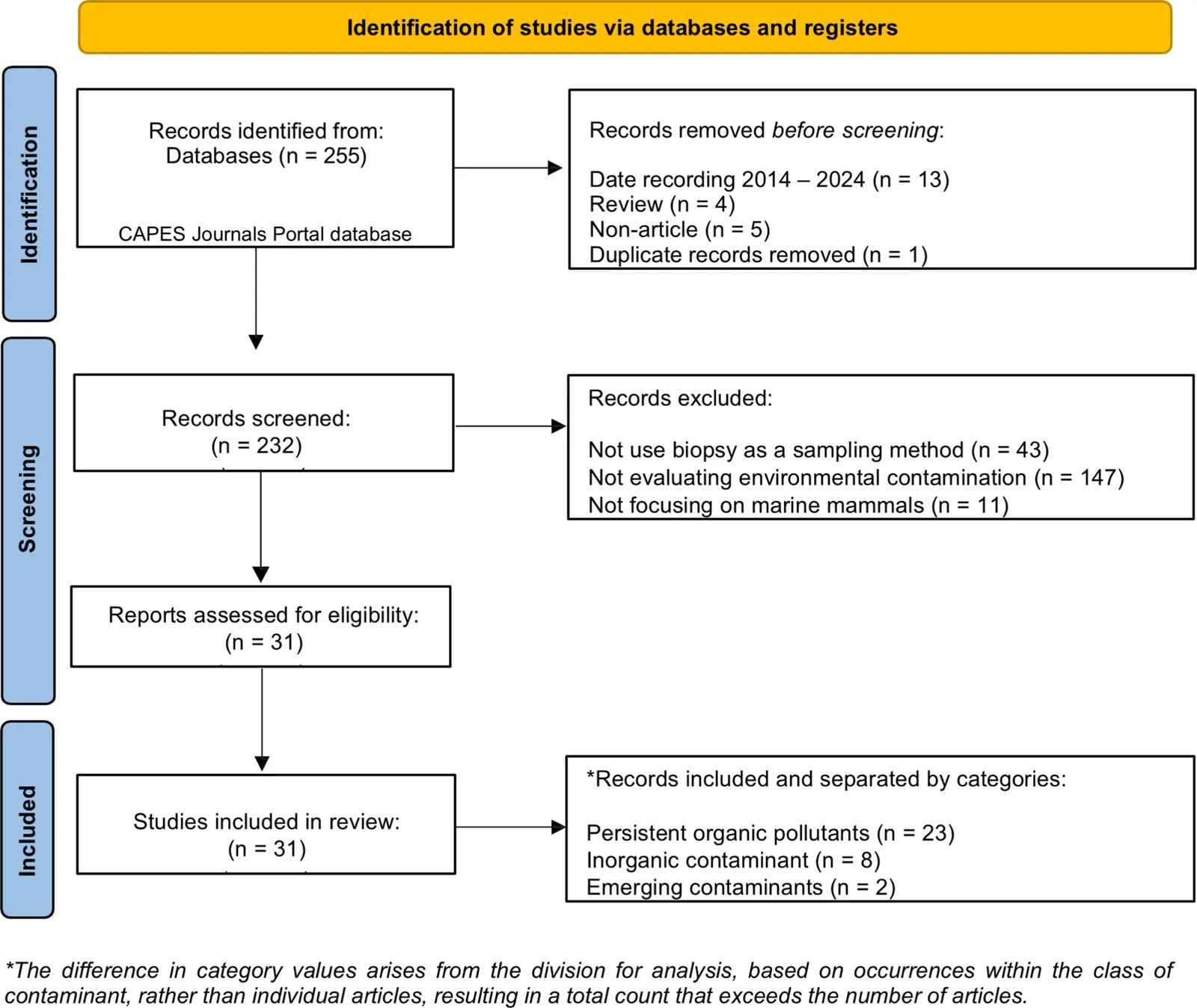
Flowchart based on the PRISMA model showing the result of the search carried out in the CAPES Periodicals Portal database (2014–2024) using the descriptors “*Biopsy*” AND “*Marine mammals*”

From the selected 323 articles, 43 were excluded because they did not use biopsy as a sampling method, 147 were excluded for not evaluating environmental contamination on individuals, and 11 were excluded for not focusing on marine mammals. The final selection included 31 articles, categorized according to the class of environmental contaminants they studied. The distribution was as follows: 23 articles focused on organic persistent pollutants (POPs), eight on inorganic contaminants, and two on emerging pollutants (Fig. 1). The sum of these categories exceeds the total number of articles because some studies (*n* = 2) investigated more than one class of contaminants. This classification reflects the frequency of occurrence of each contaminant class in the selected studies. To assess environmental impacts, the selected studies analyzed a benchmarking approach by comparing reported contaminant concentrations, toxicity thresholds, safety limits, and physiological and molecular alterations, thereby providing a comprehensive assessment of marine environmental health.

**Fig. 2:**
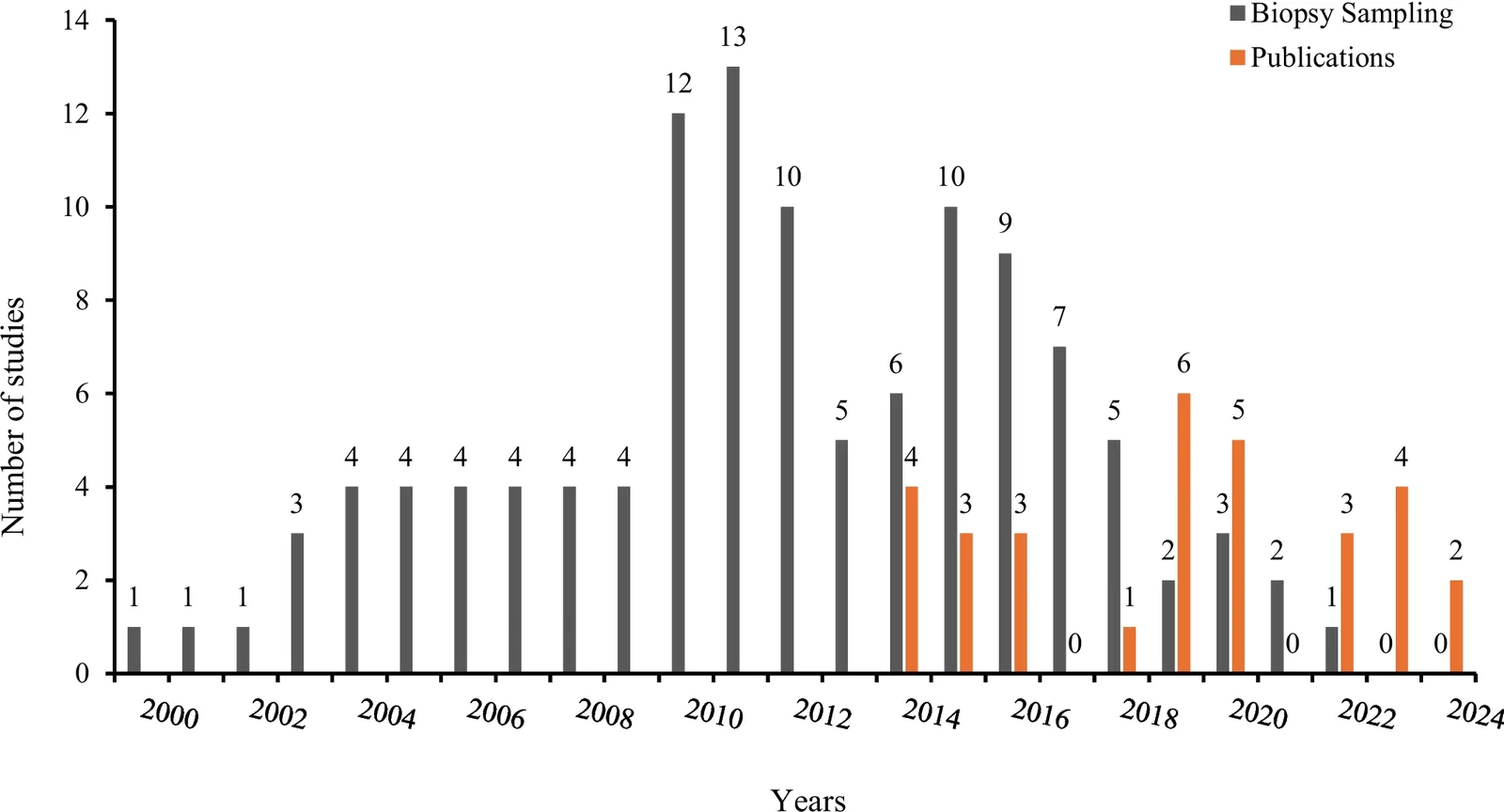
Temporal distribution of the publications included in this review, along with the annual distribution of sampling periods for the biopsies used in these articles

The temporal distribution of the 31 selected articles revealed an uneven publication pattern over the 10 years (2014–2024) and studies with a long-term sampling covered in this systematic review (Fig. 2). Regarding the number of publications, the years 2019 and 2020 showed the highest number of studies, six and five articles, respectively, representing 19.3% and 16.1% of the total publications. Four articles per year were found in 2014 and 2023 (12.9%), while 2015, 2016, and 2022 contributed three articles each (9.6%). The year 2024 had two articles (6.4%), and 2018 had only one article (3.2%). Notably, no articles were published in 2017 and 2021 (Fig. 2). However, when considering the period of active sampling, the data biopsies collection exceeds 20 years (Fig. 2). Although the number of publications peaked in 2019, an analysis of sampling periods reveals that peak fieldwork activity occurred nearly a decade earlier, with a high of 13 studies conducting sample collection in 2011. Furthermore, there is a clear distinction between publication dates and the specific years in which the biopsy sample was collected. The temporal pattern of the biopsy sampling periods for each of the studies included in this review is presented in the following sections (Persistent Organic Pollutants, Inorganic contaminants - potentially toxic metals and Emerging pollutants - plasticizers sections; Tables 1, 2, and 3), highlighting the contamination status of marine mammals and the oceans during specific timeframes. The data obtained through these biopsies provided a retrospective overview dating back to the 2000 s (Savery et al. 2014). It is important to note that the time lag between biopsy sampling and publication varies but is significant, often exceeding a decade (e.g., Savery et al. 2014; Baugh et al. 2023). This distinction is fundamental to correctly interpreting temporal trends in contaminants, since the reported levels reflect the contamination at the exact moment of sampling, influenced by the regulatory landscape and substance bans in effect at that specific time, and not necessarily the environmental conditions of the year of publication.

**Table 1:** Articles published in the CAPES Periodical Portal database (2014–2024) that used biopsy sampling of marine mammals to analyze POPs and other organic pollutants

Nº	Specie(s)	Biopsy sampling period	Sample (and quantity of)	Technique^1^	POPs^2^ concentration (min–max in ng/g)	Conclusion of contamination results	Reference
1	False killer whale (*Pseudorca crassidens*)	2009–2010	Blubber (*n *= 36)	GC-MS	(wet weight) ∑PCBs = 36–110,000	In *P. crassidens*, PCB concentrations exceeded proposed threshold levels for health effects in 84% of samples. The study showed a correlation between PCBs and CYP1A1, a biomarker of exposure	Foltz et al. (2014)^3^
2	Bottlenose dolphin (*Tursiops truncatus*)	2003–2011	Blubber (*n* = 64)	GC-MS	(lipid weight) ∑PCBs: 245–1,016.851 ∑DDTs: 189–1,252.210 ∑HCH: 0–855 ∑CLHs: 4–9689 ∑PAHs: 1394–42,577 HCB: 0–3064 Endrin: 47–60,699 Mirex:1–29,541 Heptachlor: not detected Aldrin: not detected Dieldrin: not detected ∑Endosulfan: not detected	M-PCBs represented 86% of PCBs investigated, the highest content detected. A DDT metabolite was the organochlorine pesticide detected in 87% of samples. PAHs presented low detection frequencies compared to PCBs and DDTs, but were found in all samples	García-Alvarez et al. (2014)
3	Bottlenose dolphin (*Tursiops truncatus*)	2010–2011	Blubber (*n* = 169)	GC-MS	(lipid weight) ∑PCBs: 32,700–73,400 ∑DDTs: 11,800–33,900 ∑PBDEs: 1280–4280 CLHs: 1910–5740 HCB: 30–80 Dieldrin: 100–710 Mirex: 120–590	POPs profiles varied significantly across samples collected in the northern Gulf of Mexico. PCBs showed higher means, and the coastal/estuarine regions were the contamination hotspots	Balmer et al. (2015)
4	Long-finned pilot whale (*Globicephala melas*)	2006–2013	Blubber (*n* = 49)	GC-ECD and GC-HRMS	(lipid weight) ∑PCBs: 1566–102,967 ∑DDTs: 25,114–185,209 ∑PBDEs: 205–1755 ∑HCHs: 23.4–3713 Dieldrin: 140–3860 HCB: 17.3–166.4 Aldrin: ↓ LOQ β-endosulfan: ↓ LOQ	*G. melas* exhibited the highest concentrations of ΣPCBs, ΣDDTs, and ΣPBDEs. DDTs were the most prevalent pollutants detected	Pinzone et al. (2015)^4^
	Sperm whale (*Physeter macrocephalus*)		Blubber (*n* = 61)		∑PCBs: 1581–68.475 ∑DDTs: 272–169.869 ∑PBDEs: 132–781 ∑HCHs: 8–330 Dieldrin: 278–3155 HCB: 21.4–132.6 Aldrin: ↓ LOQ β-endosulfan: ↓ LOQ		
	Fin whale (*Balaenoptera physalus*)		Blubber (*n* = 70)		∑PCBs: 2119–25,065 ∑DDTs: 1744–26,695 ∑PBDEs: 6.8–1194 ∑HCHs: not analyzed Dieldrin: not analyzed HCB: 11.9–92.2 Aldrin: ↓ LOQ β-endosulfan: ↓ LOQ		
5	Humpback whale (*Megaptera novaeangliae*)	2010–2011	Blubber and skin (*n* = 26)	GC-MS	(lipid weight) ∑PCBs: 0.6–16 ∑DDTs: 2.4–26 HCB: 6.6–67 ∑CHLs: 1.4–26 ∑HCHs: 0.4–12.2 ∑PBDEs: 0.19–12	HCB and DDTs account for 52% and 17% of total detected compounds, respectively. Males had significantly higher concentrations of HCB and DDTs than females	Das et al. (2016)^4^
6	Indo-pacific bottlenose dolphin (*Tursiops aduncus*)	2010–2011	Blubber (*n *= 32)	GC-MS	(lipid weight) ∑PCBs: 100–67,500 ∑DDTs: 26–19,345 ∑HCHs: 0.3–45 HCB: 0.2–51 ∑PBDEs: 5–1200 CHLs: not available	DDTs, PCBs, and PBDEs were the most abundant contaminants in dolphin blubbers. PCBs contributed 60% for *S. longirostris* and 64% for *T. aduncuns*. A wider range of concentrations of the pollutants investigated was found in *T. aduncus*	Dirtu et al. (2016)^4^
	Spinner dolphin (*Stenella longirostris*)		Blubber (*n* = 21)		∑PCBs: 30–2170 ∑DDTs: 49–1550 ∑HCHs: 0.5–65 HCB: 1.5–29 ∑PBDEs: 10–120 CHLs: not available		
7	Fin whale (*Balaenoptera physalus*)	2012–2013	Blubber (*n* = 40)	GC-ECD	(lipid base) ∑PCB: mean 52,898.9 ∑DDT: mean 38,754.5 HCB: mean 136.3	Whales sampled in the Mediterranean Sea were more contaminated by PCBs than those in the Gulf of California. PCBs were the principal contaminants detected in these whales	Fossi et al. (2016)^5^
8	Bottlenose dolphin (*Tursiops truncatus*)	2010–2012	Blubber (*n* = 116)	GC-MS	(lipid base) ∑PCBs: ~ 30,000–50,000 ∑DDTs: ~ 15,000–25,000 HCB: ~ < 1,000 ∑PBDEs: ~ < 5000 ∑CHLs: ~ 2000–8000 Dieldrin: < 1000 Mirex: < 2000	Dolphins from the southeastern U.S. coast exhibited higher levels of ∑POPs compared to those from the northern Gulf of Mexico, underscoring the high contamination of those cetaceans by PCBs	Neely et al. (2018)^6^
9	Bottlenose dolphin (*Tursiops truncatus*)	2011–2012	Blubber (*n* = 70)	GC-MS	(lipid base) ∑PCBs: 900–93,000 ∑DDTs: 100–89,000 ∑PBDEs: 100–3900 ∑CHLs: 0–4800 HCB: 0–100 Dieldrin: 0–500 Mirex: 0–500	*T. truncatus* exhibited the highest contamination levels, with higher concentrations of POPs found in males compared to females (except for one female, which never had calves). Among POP classes, PCBs and DDTs were the most abundant	Balmer et al. (2019)^4^
	Atlantic spotted dolphin *Stenella frontalis*		Blubber (*n* = 4)		∑PCBs: 400–45,000 ∑DDTs:0–26,000 ∑PBDEs: 0–4900 ∑CHLs: 0–3900 HCB: 0 Dieldrin: 0–100 Mirex: 0–200		
10	Bottlenose dolphin (*Tursiops truncatus*)	2015–2016	Blubber (*n* = 62)	GC-MS	(lipid base) ∑PCBs: 57–46,000 ∑DDTs: 8.5–62,000 ∑BDEs: < 2.1–800 ∑CHLs: < 2–2200 HCB: < 1.2–76 Dieldrin: < 1.2–170 Mirex: < 2–130	DDT was the principal xenobiotic, and the males exhibited higher contents of other POP classes compared to females	Galligan et al. (2019)^4^
11	Bottlenose dolphin (*Tursiops truncatus*)	2011–2017	Blubber (*n* = 31)	GC-μECD	(lipid weight) ∑PCBs: 4130–293,000 ∑DDTs: 32,900–300,000 HCB: 30–220	Males exhibited higher PCB concentrations than females. The mean concentrations of Σ25PCB were above the established toxicity thresholds for physiological and reproductive effects in marine mammals, with 87.5% of dolphins exceeding the lower threshold	Genov et al. (2019)^4^
12	Bottlenose dolphin *(Tursiops* *truncatus gephyreus)*	2015–2016	Blubber (*n* = 17)	GC-MS and GC-ECD	(lipid weight) ∑PCBs: 695.97–51,491.59 ∑DDTs: 99.49–17,267.56 ∑PBDEs: 0.24–590 ∑CHLs: 0.21–257.94 HCB: 0.24–181.99 Mirex: 0.24–567.55	PCBs were the most prevalent pollutants in the estuarine region. POPs concentrations were generally consistent between the populations, but dolphins from the Patos Lagoon Estuary accumulated PCB concentrations twice as high as those from the Laguna Estuary	Righetti et al. (2019)^4^
13	Bottlenose dolphin (*Tursiops truncatus*)	2018	Blubber (*n* = 11)	GC-MSD	(lipid weight) ∑PCBs: 300–5400 ∑DDTs: 120–7000 ∑PBDEs: 24–641 ∑CHLs: 10–100 Dieldrin: 4–150	The predominant pollutants were DDTs, accounting for 47% of ∑POPs, with higher concentrations found in adult males. Females exceeded immunotoxic thresholds for PCBs	Alava et al. (2020)^4^
14	Cuvier’s beaked whale (*Ziphius cavirostris*)	2014–2015	Skin (*n* = 22)	GC-HRMS	(lipid weight) ∑PCBs: 11,820–28,170 ∑PBDEs: 260–600	PCB concentrations were higher than PBDEs. PCBs exceeded thresholds for physiological effects and reproductive risk, as well as immunosuppression levels	Baini et al. (2020)^4, 5^
15	Humpback whale (*Megaptera novaeangliae*)	2014–2017	Blubber (*n* = 148)	GC-MS	(lipid weight) ∑PCBs: 0.6–22.7 ∑DDTs: 0.4–153.0 ∑PBDEs: 0.3–0.9 ∑CHLs: 0.7–16.9 HCB: 6.1–127.0 ∑HCHs: 0.3–4.9	HCB was the contaminant found in the highest concentrations in both regions. Correlation between DDT concentrations and trophic position was significant in Ecuadorian whales. POPs concentrations were higher in males than in females	Remili et al. (2020)^4^
16	Blue whale (*Balaenoptera musculus*)	2014–2018	Blubber (*n* = 18)	GC-MS	(lipid weight) ∑PCBs: 55–334 ∑DDTs: 40–669 ∑PBDEs: 2–17 ∑CHLs: 33–189 HCB: 12–82 ∑HCHs: 4.6–22 Mirex: 0.9–6.2 Toxaphene: 35–288	POPs concentrations were higher in males, and *B. physalus* exhibited greater contamination levels than *B. musculus* due to their higher trophic position. PCBs were the most prevalent pollutants to both species, with emphasis on PCB153 and PCB138 congeners	Tartu et al. (2020)^4^
	Fin whale (*Balaenoptera physalus*)		Blubber (*n* = 12)		∑PCBs: 79–522 ∑DDTs: 113–681 ∑PBDEs: 4.7–94 ∑CHLs: 49–323 HCB: 35–139 ∑HCHs: 7.8–28 Mirex: 2.5–8.6 Toxaphene: 138–418		
17	Grey whale (*Eschrichtius robustus*)	2003–2017	Blubber (*n* = 120)	GC-McaS	(lipid weight) ∑PCBs: 0–1,940 ∑DDTs: 0–2,630 ∑PBDEs: 0–350 ∑CHLs: 0–460 HCB: 0–370 ∑HCHs: 0–270 Mirex: < 0.53–1.6 Endosulfan I: ↓ LOQ	PCBs and DDTs were detected in higher concentrations. Adult males exhibited higher POPs levels than females. Calves showed high POPs due to maternal transfer during lactation	Hayes et al. (2022)^4^
18	Beluga whale (*Delphinapterus leucas*)	2016–2018	Blubber (*n* = 42)	GC/MS-ECNI	(wet weight) ∑PBDEs: 41.5–666,000 ∑HFRs: 0.16–39	The PBDEs were the predominant contaminants detected in males of *D. leucas* from the St. Lawrence Estuary	Jia et al. (2022)
19	Orca (*Orcinus orca*)	2015	Blubber (*n* = 7)	GC-LRMS	(lipid weight) ∑PCBs: 420–3970 ∑DDTs: 501–3550 ∑PBDEs: 18.4–138 HCB: 74.3–198 ∑HCHs: 15.1–56.40 DP: 0.0073–0.0741	Among the POPs investigated, DDTs were found in higher amounts. Despite the low levels of DP, it was detected in all specimens, suggesting limited trophic transfer and potential degradation in *O. orca*	Panti et al. (2022)^4^
20	Humpback whale (*Megaptera novaeangliae*)	2004–2012	Blubber (*n* = 36)	GC-MS	(lipid weight) ∑PCBs: 280–12,000 ∑DDTs: 100–5100 ∑PBDEs: 28–2000 ∑CHLs: 66–2000 ∑HCHs: 6.2–110	Concentrations of POPs varied over age class, with juveniles showing the highest levels. PCBs were the most abundant xenobiotics found. Calves had the highest contaminant concentrations, likely due to maternal transfer	Baugh et al. (2023)^4^
21	Orca (*Orcinus orca*)	2020	Skin (*n* = 5)	ddPCR and RNA seq	PCB, DDT, and CHL; Concentrations not quantified	*O. orca* cell cultures exposed to POPs mixtures exhibited early signs of cellular stress, compromised detoxification systems, and systemic cellular dysregulation	Bjørneset et al. (2023)^7^
22	Northern Elephant Seal (*Mirounga angustirostris*)	2016–2017	Blubber (*n* = 35)	GC-MSD	(dry weight) ∑PCBs: 73.3–4,487.6 ∑DDTs: 429–28,744.4 ∑CHLs: 51.2–703.30 HCB: 7.7–92.20	∑DDTs were the most abundant POPs across all individuals studied. Adults showed higher concentrations, suggesting age bioaccumulation. The greater contamination variability in females can be attributed to foraging grounds and niche segregation	Muntaner-López et al. (2023)^4^
23	Orca (*Orcinus orca*)	2013–2021	Blubber (*n* = 33)	GC-HRMS	(lipid weight) ∑PCBs: 4.08–700.48 ∑DDTs: 2.67–876.60 ∑CHLs: 1.14–349.50 β-HCH: 0.0059–4.81 Chlorobenzenes: 0.037–17.51 Toxaphene: 0.56–261.13 Mirex: 0.082–9.90 ∑PCNs: 0.016–0.23	DDTs were detected in the highest concentrations. *O. orca* sampled in the Canadian Arctic exhibited higher POPs concentrations, with potential health risk to humans	Desforges et al. (2024)^4^

^1^*GC-MS*, gas chromatography-mass spectrometry; *GH-HRMS*, gas chromatography-high resolution magnetic sector mass spectrometry; *GC*, gas chromatography, *GC-ECD*, gas chromatography with electrons capture detector; *GC-μECD*, gas chromatography with micro electron capture detector; *GC-MSD*, gas chromatography-mass selective detector; *GC-HR*, gas chromatography-high resolution; *GC-LRMS*, gas chromatography low resolution mass spectrometry

^2^*PCBs*, polychlorinated biphenyls; *DDTs*, dichlorodiphenyltrichloroethanes; *CHLs*, chlordanes; *HCB*, hexachlorobenzene; *PAHs*, polycyclic aromatic hydrocarbons; *PBDEs*, polybrominated diphenyl ethers; *HCH*, hexachlorocyclohexane; *MeO-PBDEs*, methoxylated polybrominated diphenyl ethers; *HFR*, halogenated flame retardants; *PCNs*, polychlorinated naphthalene; *BDEs*, brominated diphenyl ethers

^3^The study also included nine more species of odontocetes, but the contamination analyses were only to *P. crassidens*

^4^The study evaluated males and females

^5^The study presents only mean concentration values

^6^The study presents concentration values only in a graphic, so the range presented here is an estimate (~)

^7^The study evaluated the effects of exposure

↓LOQ: below the limit of quantification

**Table 2:** Articles published in the CAPES Periodical Portal database (2014–2024) that used biopsy sampling to identify inorganic contaminants like potentially toxic metals in marine mammals

Nº	Specie(s)	Biopsy sampling period	Sample (and quantity of)	Inorganic contaminant^1^	Technique^2^	Concentration values found (min – max in ng/g)	Contamination results	Reference
1	Burrunan dolphin (*Tursiops australis*)	2004–2008	Blubber (*n* = 20)	Hg, Pb, As, and Se	ICP-MS and ICP-OES	Hg: 320–4200 Pb: 760–12,000 As: < 100–380 Se: 520–3900	Dead dolphins had higher Hg contamination than live animals. Mercury in dolphins’ prey suggests dietary bioaccumulation	Monk et al. (2014)
2	Sperm whale *(Physeter macrocephalus)*	2000–2005	Skin (*n* = 342)	As	ICP-MS	100–15,600	As levels varied by region and gender, males showed higher concentrations than females. A slight positive correlation was observed between arsenic and chromium levels	Savery et al. (2014)^4^
3	Fin whale (*Balaenoptera* *physalus)*	2010–2012	Skin (*n* = 9)	Cr	ICP-AES	1710–19,600	All samples presented Cr contamination. Cr, particularly in particulate form, poses significant health risks to fin whales	Wise et al. (2015)^4^
4	Humpback whale (*Megaptera novaegliae*)	2010–2012	Skin (*n* = 33)	Al, Cr, Ni, Fe, Mg, Zn, Ti, Se, As, Cd, Co, Pb, Hg, U, Sb, Be, Au, Sr, Cu, Mn, Ba, Sn, V, Ag, and Se	ICP-MS	Mg: 83,590–507,57 Fe: 49.11–228.39 Be and U: 10–20	Humpback whales showed the highest levels of potentially toxic metals among the three whale species analyzed. Mg, Fe, Se, Zn, and Ni were the main metals detected in whale tissues	Wise et al. (2019)^3^
	Fin whale (*Balaenoptera physalus*)		Skin (*n* = 9)			Zn: 13,800–45,250 Ni: 10,680 Hg: 10–80 Cd: 10–70 Zn: 16,030		
	Minke whale (*Balaenoptera acutorostrata*)		Skin (*n* = 1)			Se: 5530 Other metals: < 700		
5	Fin whale (*Balaenoptera physalus*)	2010–2014	Skin (*n* = 67)	Hg	AAS	262–1363	Hg analysis revealed distinct contents for the three cetacean species investigated. *G. melas* showed the highest Hg concentrations. The study suggests that these variations are likely influenced by diet and habitat differences	Pinzone et al. (2019)^4^
	Long-finned pilot whale (*Globicephala melas*)		Skin (*n* = 36)			17,235–49,496		
	Sperm whale *(Physeter macrocephalus)*		Skin (*n* = 29)			4235–18,080		
6	Grey whale *(Eschrichtius robustus)*	2011	Skin (*n* = 10)	Hg	DMA	< 8.0–98.92	Hg concentration varied across epidermal layers in eastern gray whales, with significant differences in both wet and dry weight measurements	Lian et al. (2020)^4^
7	Southern elephant seal (*Mirounga leonina*)	2015–2016	Skin (*n* = 60)	Hg	AAS	145–1915	Females had significantly higher concentrations than males, suggesting contamination linked to foraging strategies	Barragán-Barrera et al. (2023)^4^
8	Bottlenose dolphin (*Tursiops truncatus*)	2018	Skin (*n* = 13)	Hg	AAS and DMA	1920–3630	The estimated proportion of Hg in the skin was around 70%, indicating potential bioaccumulation	Alava et al. (2020)^4^

^1^*Hg*, mercury; *T-Hg*, total mercury; *As*, arsenic; *Pb*, lead; *Cr*, chromium; *Ni*, nickel; *Fe*, iron; *Mg*, magnesium; *Zn*, zinc; *Al*, aluminum; *Be*, beryllium; *U*, uranium; *Cd*, cadmium; *Sb*, antimony; *Au*, gold; *Ba*, barium; *Sn*, tin; *Co*, cobalt; *V*, vanadium; *Ag*, silver; *Sr*, strontium; *Cu*, copper; Mn, manganese; *Ti*, titanium; *Se*, selenium

^2^*ICP-MS*, inductively coupled plasma mass spectrometer; *ICP-OES*, inductively coupled plasma optical emission spectroscopy, *ICP-AES*, inductively coupled plasma atomic emission spectrometry; *AAS*, atomic absorption spectrometry; *DMA*, direct mercury analyzer

^3^The study presents only mean concentration values

^4^The study evaluated males and females

**Table 3:** Articles published in the CAPES Periodical Portal database (2014–2024) reporting the use of biopsy sampling to identify emerging pollutants like plasticizers in marine mammals

Nº	Specie(s)	Biopsy sampling period	Sample (and quantity of)	Emerging Pollutant^1^*	Technique^2^	Concentrations values found (min–max in ng/g)	Contamination results	Reference
1	Fin whale (*Balaenoptera physalus*)	2012–2013	Skin (*n* = 40)	MEHP	GC-ECD	(lipid base) MEHP: 40.2–65.5	Whales in the Mediterranean Sea exhibited higher MEHP levels compared to the Gulf of California, highlighting significant regional contamination linked to anthropogenic pollution	Fossi et al. (2016)^3^
2	Short-finned pilot whale (*Globicephala macrorhynchus*)	2017–2022	Blubber (*n* = 45)	DEHP DMP, DEP,, DIBP, DBP, BBP, DNOP	GC-MS	(wet weight) DEHP: 0–93.63 DMP: 0–25.98 DEP: 5–404.30 DIBP: 0–19.41 DBP: 0–284.11 BBP: 0–10.41 DNOP: 0–13.37	All individuals have at least one phthalate detected. DEHP was the majoritarian ester detected in dolphin samples, as DEP and DBP were found in higher amounts in pilot whales	Sambolino et al. (2024)^4^
	Bottlenose dolphin (*Tursiops truncatus*)		Blubber (*n* = 39)			DEHP: 127.30–4697.34 DMP: not detected DEP: 0–38.94 DIBP: 0–92.70 DBP: 0–717.59 BBP: 5–57.11 DNOP: not detected		

^1^*DEHP*, di-(2-ethylhexyl) phthalate; *MEHP*, mono-(2-ethylhexyl) phthalate; *DMP*, dimethyl phthalate; *DEP*, diethyl phthalate; *DIBP*, di-isobutyl phthalate; *DBP*, dibutyl phthalate; *BBP*, benzyl-butyl phthalate; *DNOP*, di-n-octyl phthalate

^2^*GC-ECD*, gas chromatography with electron capture detection; *GC-MS*, gas chromatography-mass spectrometry

^3^The study presents only mean concentration values

**Fig. 3:**
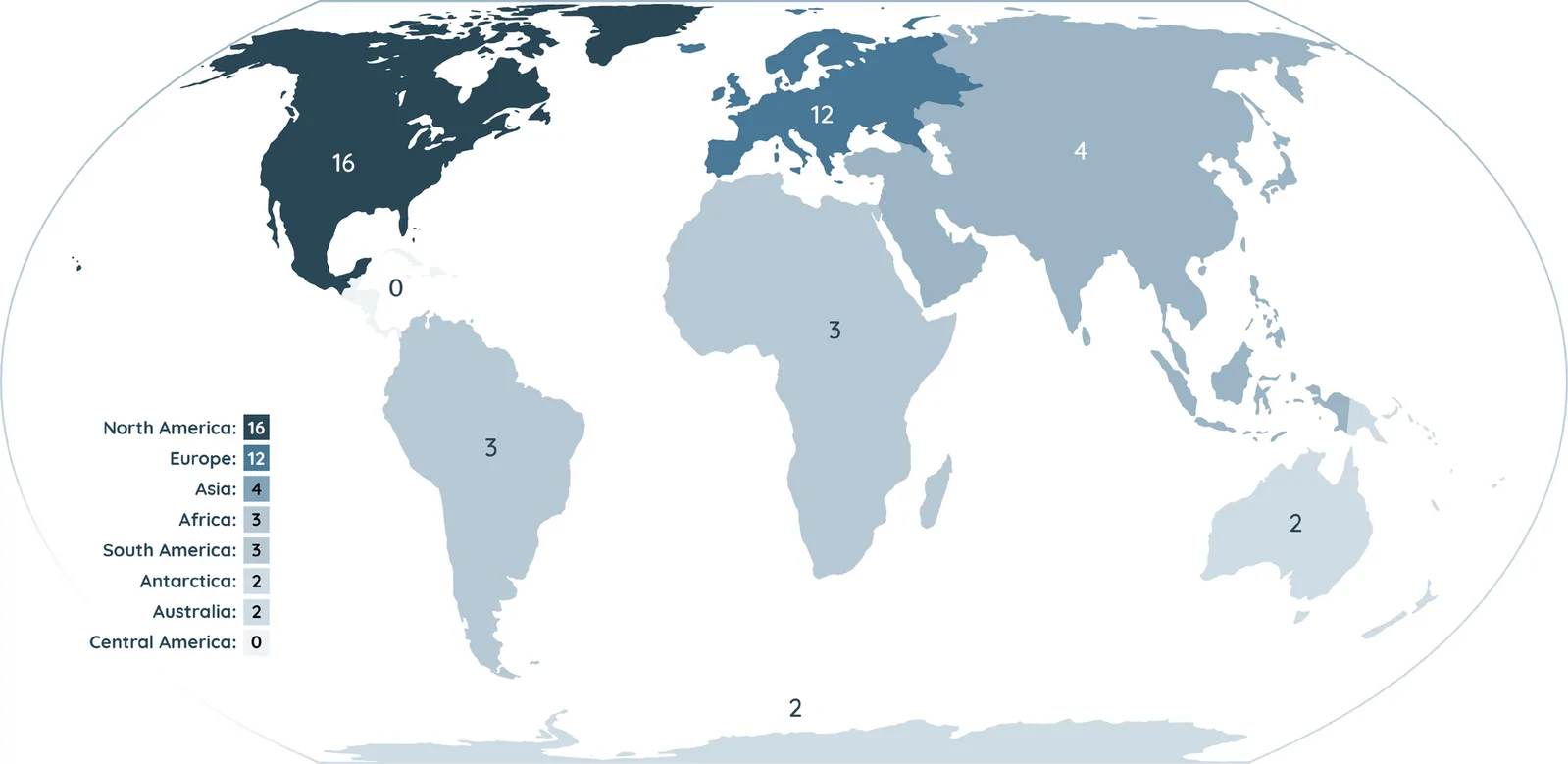
Distribution of studies by continent using the descriptor “*Biopsy*” AND “*Marine mammals*” in the CAPES Platform. The total number of publications exceeds the number of articles included in the review because some studies evaluated animals across multiple geographic regions

The analysis of the geographical distribution revealed that the number of continental occurrences (*n* = 42) exceeded the total number of articles (*n* = 31), as some studies were conducted across multiple continents (Fig. 3). North America showed the highest concentration of research (*n* = 16), representing 38.1% of the studies. Europe followed with 12 articles (28.6%), while Asia accounted for four studies (9.5%). Africa and South America each contributed three publications (7.1% each), whereas Antarctica and Australia contributed two articles each (4.8% each). This distribution likely reflects the greater availability of research infrastructure, funding, and technological resources in North America and Europe, as well as the higher diversity and abundance of marine mammals in those regions. Additionally, North America and Europe have historically been at the forefront of marine mammal research and environmental monitoring, which may explain their dominance in this research field (Tolochko and Vadrot 2021; Vis et al. 2024).

The analysis of biopsied tissues revealed that blubber was the most frequently sampled material, appearing in 23 articles (~74%), and skin was found in 12 articles (38.7%). The use of distinct tissues in some studies explains why the total number of tissue occurrences (*n* = 35) exceeds the number of articles (*n* = 31). The predominance of blubber as a sampling target is likely due to its vital role in the physiology of marine mammals, particularly in thermoregulation and energy storage (Jefferson et al. 2015). Blubber is also known to accumulate lipophilic environmental contaminants, such as persistent organic pollutants (POPs), making it a highly representative tissue for studying environmental contamination (Baugh et al. 2023).

**Fig. 4:**
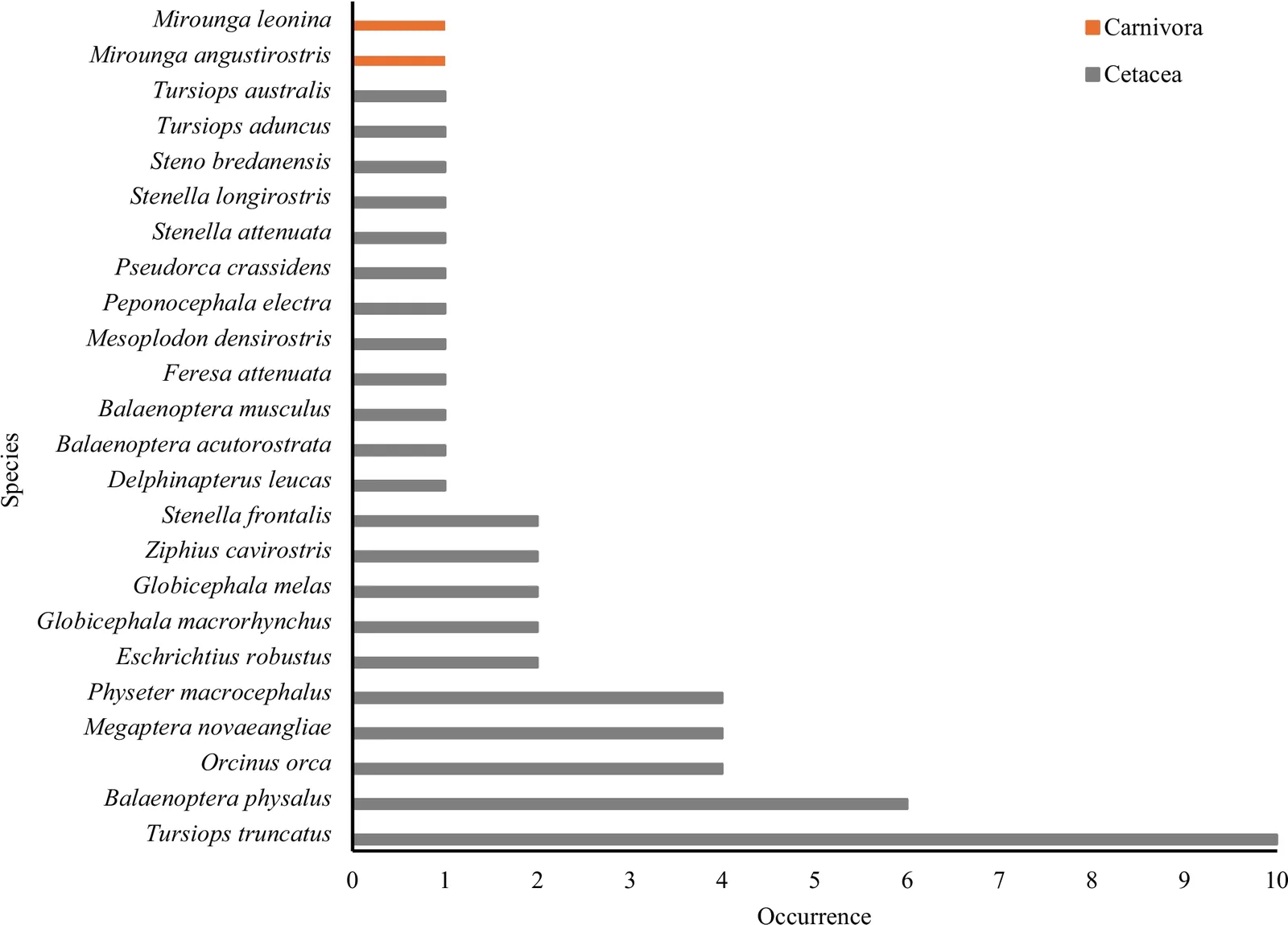
Number of publications with species and their taxonomic order using the descriptor “*Biopsy*” AND “*Marine mammals*” in the CAPES Journals Platform (2014–2024)

The studies focused on a diverse range of marine mammal species (Fig. 4), with a clear predominance of cetaceans, especially bottlenose dolphins (*Tursiops truncatus)*, the most frequently studied species, appearing in 11 articles (35.8%). Other cetaceans, such as fin whale (*Balaenoptera physalus)*, humpback whale (*Megaptera novaeangliae*), and sperm whale (*Physeter microcephalus*), were also well-represented, each appearing in four articles. The Carnivora order, pinnipeds, was less represented in this review, with only two articles focusing on the elephant seal species, Northern elephant seal (*Mirounga angustirostris*) and Southern elephant seal (*Mirounga leonina*). The predominance of cetaceans in the studies may be attributed to their widespread distribution and the extensive use of biopsy sampling as a standard non-invasive technique for analyzing genetics, contaminants, fatty acids, and hormones (Graham et al. 2021; Krahn et al. 2023). Additionally, biopsy sampling in cetaceans may be considered less stressful than that in seals because it requires more contact, such as physical handling, restraint, or sedation (Bennet et al. 2012; Champagne et al. 2012), with contaminant analyses being more common in stranded pinnipeds (Bertolazzi et al. 2026).

**Fig. 5:**
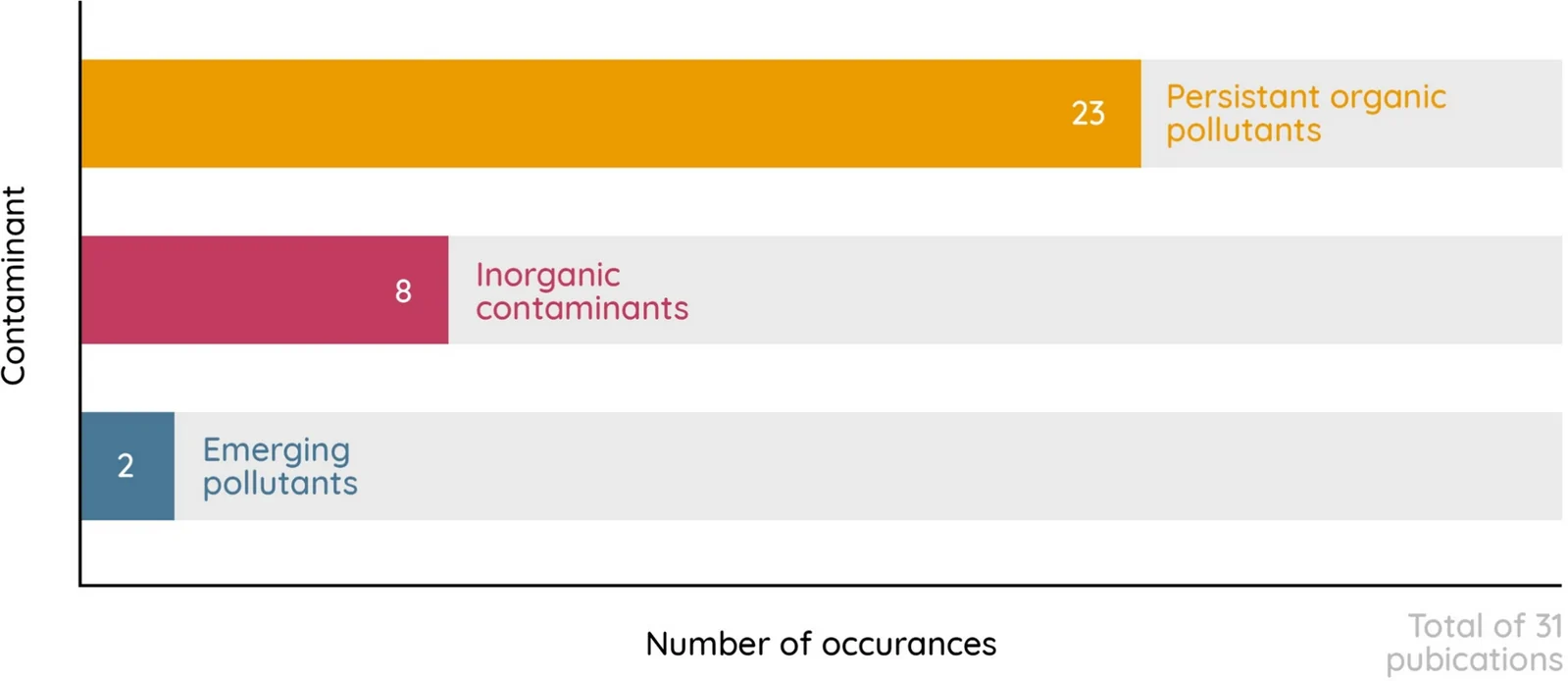
Occurrence of contaminants identified in the studies using the descriptors “*Biopsy*” AND “*Marine mammals*” in the CAPES Periodicals Platform (2014–2024)

The identified contaminant classes included persistent organic pollutants (POPs), with 23 occurrences, potentially toxic metals (inorganic contaminants), with eight occurrences, and emerging pollutants, including plastics and plasticizers, with two occurrences (Fig. 5). As some studies investigated more than one contaminant class, the total number of occurrences exceeded the number of articles selected for the review.

The results are presented in descending order of contaminant occurrence, from the most studied (POPs) to the least studied (emerging pollutants). For each class, specific contaminants, sampling methods, levels, and their implications for the health of marine mammals, ecosystems, and humans are described. This approach provides a comprehensive understanding of how biopsy studies contribute to research on environmental contamination and highlights the interconnectedness of animals, the environment, and human health. Only contamination results from live biopsied animals were considered, though studies comparing deceased and living individuals were also included for a more in-depth discussion.

### Persistent organic pollutants (POPs)

Twenty-three articles were identified in the category of POPs (Table 1), with publications spanning from 2014 (*n* = 2), 2015 (*n* = 2), 2016 (*n* = 4), 2018 (*n* = 1), 2019 (*n* = 4), 2020 (*n* = 4), 2022 (*n* = 3), 2023 (*n* = 3), and 2024 (*n* = 1). The pollutants analyzed in the selected articles, ordered by frequency of occurrence, were as follows: polychlorinated biphenyls (PCBs; *n* = 22), dichlorodiphenyltrichloroethane (DDTs; *n* = 21), hexachlorobenzene (HCB; *n* = 16), polybrominated diphenyl ethers (PBDEs; *n* = 15), chlordane (CHLs; *n* = 15), hexachlorocyclohexanes (HCHs; *n* = 10), dieldrin (*n* = 8), mirex (*n* = 10), endosulfan (*n* = 3), toxaphene (*n* = 2), chlorobenzenes (*n* = 1) polychlorinated naphthalene (PCNs; *n* = 1), and halogenated flame retardants (HFRs; *n* = 1).

The analysis of the 23 studies selected in this systematic review addressing POPs contamination reveals exposure patterns driven by geographic and ecological factors, such as anthropogenic influences. Among POPs-which are carbon-based chemicals characterized by persistence, bioaccumulation, and toxicity- PCBs and DDTs emerged as the most frequently studied contaminants (Table 1), being the primary focus of long-term monitoring efforts in both hemispheres The studies compiled within this category exhibit a primary concentration in the Northern Hemisphere (*n* = 10), in zones such as the Northwest Atlantic and southeastern US coastlines (Balmer et al. 2015; Neely et al. 2018; Galligan et al. 2019; Baugh et al. 2023), the semi-enclosed basins of the Mediterranean and Adriatic Seas (Pinzone et al. 2015; Fossi et al. 2016; Baini et al. 2020; Genov et al. 2019), and high-latitude environments in the Arctic and sub-Arctic waters (Bjørneset et al. 2023; Desforges et al. 2024). In contrast, POPs analyses, utilizing biopsies as sampling, across the Southern Hemisphere remain fragmented and scarce (*n* = 4), represented by localized monitoring efforts in the southwestern Indian Ocean (Dirtu et al. 2016; Das et al. 2016), subtropical estuarine areas in South America (Righetti et al. 2019), and remote feeding grounds in Antarctica (Panti et al. 2022).

The persistent organic pollutants include pesticides, industrial compounds, and byproducts such as dioxins generated during combustion processes, which contaminate soil, water, and air, affect biodiversity and travel long distances through the atmosphere, even reaching remote regions like the poles (Hung et al. 2016; Hao et al. 2019; Jones 2021). Their impacts include risks to human health, such as carcinogenic effects, whether through environmental exposure or diet (Linares et al. 2010). To mitigate harm, the Stockholm Convention regulates the elimination or restriction of POPs and mandates that countries adopt control measures related to production, import, export, use, and disposal of substances listed in its annexes (Stockholm Convention 2025). The global hazard posed by those pollutants demands continuous monitoring, as they threaten future generations due to their durability and widespread dispersal.

DDTs and PCBs stand out as the primary contaminants in all studied regions. Both were banned in the 1970 s and 1980 s, respectively, yet their continued presence in marine mammals reflects their historical intensive use and resistance to degradation. Despite being banned, DDT’s use continued in some regions such as Mexico and South Africa for malaria control, extending into the late 1990 s and early 2000 s (Bouwman et al. 2011). This ongoing application presents a paradox because DDT was considered “good” for saving lives, but “not safe” due to its known health and environmental consequences. The prevalence of these POPs highlights not only the chemical stability of these compounds, which persist in the environment for decades, but also their capacity for biomagnification across trophic chains. For example, in False killer whales from Hawaii, 84% of individuals exceeded safety thresholds for ∑PCBs, with average concentrations reaching 30,000 ng/g l.w. (Foltz et al. 2014). Meanwhile, Adriatic dolphins exhibited p,p′-DDE (a DDT metabolite) comprising 90% of ∑DDTs (Genov et al. 2019). These findings underscore that, despite international bans, the toxic legacy of historical pesticides and industrial products continues to pressure marine ecosystems.

The geographic distribution of POPs contamination in marine mammals reflects a combination of historical industrial activity, environmental processes, and ecological dynamics. The Mediterranean Sea emerged as the most studied region, with 39.1% (9/23) of articles focusing on its waters, driven by a historical hub for PCB and DDT use, restricted water circulation limiting pollutant dispersion, and coastal urbanization (Marsili et al. 2018; Fossi et al. 2016; Castro-Jiménez et al. 2021). However, the Arctic region also shows alarming levels of cumulative POPs, e.g., killer whales with POPs = 216 mg/kg l.w. (216,000 ng/g l.w.), while the Gulf of Mexico has localized dolphin concentrations as high as those in the Mediterranean Sea, e.g., PCBs = 82.5 μg/g l.w. (82,500 ng/g l.w.) and DDTs = 164 μg/g l.w. (164,000 ng/g l.w.) (Balmer et al. 2015, 2019). This phenomenon reflects not only local contamination (e.g., historical pesticide uses in Europe) but also the global transport of POPs, which volatilize in tropical climates and are deposited in polar regions (Kallenborn et al. 2015). Simultaneously, coastal urbanization and industrial discharge remain significant drivers of chronic exposure, particularly for estuarine and resident populations near anthropogenic hotspots (Balmer et al. 2015, 2019; Neely et al. 2018; Alava et al. 2020). In South America, a clear example of this coastal contamination was evidenced by Righetti et al. (2019). The study observed that isolated populations of bottlenose dolphins (*T. truncatus gephyreus*) that exhibit high site fidelity to coastal habitat face significant risks due to chronic exposure to POPs, particularly in areas adjacent to industrial complexes, such as Patos Lagoon Estuary in southern Brazil. These dolphins accumulated ∑PCBs concentrations twice as high (up to 51,491 ng/g l.w.) compared to those from the adjacent less impacted waters (Righetti et al. 2019).

Factors such as sex, age, and species often prove to be significant determinants of contaminants in the accumulation. In this sense, a notable pattern was extracted from the selected articles, where 82.6% (19/23) of the studies evidenced higher POP accumulation in males compared to females, as seen in Adriatic dolphins, for instance, where p,p′-DDE concentrations were five times higher in males (Genov et al. 2019). In fact, this pattern has been observed across multiple species, including large cetaceans such as blue whales (*Balaenoptera musculus*) (Tartu et al. 2020), medium and smaller odontocetes such as killer whales (*Orcinus orca*) (Desforges et al. 2024), bottlenose dolphins (*T. truncatus gephyreus*) (Genov et al. 2019), and pinnipeds (Muntaner-Lopez et al. 2023), with several studies emphasizing a significant decline in female burdens following successive reproductive cycles (Das et al. 2016; Remili et al. 2020; Hayes et al. 2022). This disparity between genders is primarily attributed to a reduction in contaminant loads in females due to maternal offloading, rather than an increase in males. During gestation, POPs such as PCBs, DDTs, and PBDEs are transferred from mother to fetus, accumulating in fetal tissues, and after birth, the calves continue to receive POPs through lactation, leading to increasing concentrations in their serum and tissues that often exceed maternal levels, particularly for certain POP classes (Andvik et al. 2023; Lee et al. 2023; Noren et al. 2024). A similar pattern seems to exist in pinnipeds (Hitchcock et al. 2017), but the study in pinnipeds presented in this review is insufficient for robust conclusions.

Another notable result concerns the juvenile subjects that exhibited high contamination levels, suggesting that accumulation occurs before sexual maturity. It has been suggested that elevated contaminant amounts in juveniles in comparison to adults, as observed in humpback whales (ΣPCBs = 12,000 ng/g l.w., Baugh et al. 2023) and Northern elephant seal (*Mirounga angustirostris*) (ΣDDTs = 2304 ng/g d.w., Muntaner-Lopez et al. 2023), result from their developing bodies that seem to be more susceptible to accumulation and higher exposure rates during early life stages, while post-weaning lipid redistribution and metabolic excretion likely reduce adult burdens (Hayes et al. 2022; Xuereb et al. 2023).

Trophic position strongly predicted POP exposure, with apex predators exhibiting 10–40 × higher concentrations than lower-trophic species. For example, sperm whales (*Physeter macrocephalus*) (ΣPCBs = 22,849 ng/g l.w.) and killer whales (*Orcinus orca*) (ΣPOPs = 216,000 ng/g. l.w.) accumulated significantly more contaminants than krill-feeding fin whales (*Balaenoptera physalus*) (ΣPCBs = 572 ng/g l.w.) (Pinzone et al. 2015; Desforges et al. 2024). This biomagnification aligns with dietary preferences: sperm whales (*Physeter macrocephalus*) consume lipid-rich cephalopods, while killer whales (*Orcinus orca*) prey on contaminated seals and cetaceans (Tartu et al. 2020). Filter feeders like humpback whales (*Megaptera novaeangliae*) showed moderate contamination except in hotspots (e.g., Mediterranean Sea: ΣDDTs = 10,477 ng/g l.w., Fossi et al. 2016), underscoring habitat-specific risks. Killer whales (*Orcinus orca*), which prey on lipid-rich animals (seals, cetaceans), and sperm whales (*Physeter macrocephalus*), which consume bentho-pelagic prey, reflect diet-driven contamination pathways. Species feeding at higher trophic levels, or those with diets rich in contaminated prey, tend to have higher concentrations of POPs, such as PCBs and DDTs in their tissues (Dirtu et al. 2016; López-Berenguer et al. 2023).

POPs disrupt multiple metabolic processes in marine mammals, particularly those related to energy and fat metabolism. For example, in gray seal pups, higher POPs burdens decreased glucose use and altered lactate production and lipolysis. These changes can impair the ability to store and mobilize fat, which is vital for young animals’ growth and survival (Montaño et al. 2013; Robinson et al. 2018; Bjørneset et al. 2023). At the molecular level, 52.17% (12/23) of the selected studies documented that POPs showed disrupted detoxification, endocrine, and neurological systems. In parallel, to complement traditional in vitro assays, Bjorneset et al. (2023) conducted a toxicological assessment using primary skin fibroblasts derived from biopsy samples from two killer whale (*Orcinus orca*) individuals. The authors tested a mixture of the 10 most abundant POPs (including p,p′-DDE and PCBs) at environmentally relevant concentrations, to evaluate a realistic context before cell isolation. The bioassays utilized exposure treatments at 0.1× (1.16 μM), 1× (11.57 μM) and 10× (115.7 μM). The 1× level replicated the median value previously detected in blubber of free-ranging individuals from Norwegian populations by Andvik et al. (2020). The study of Bjorneset revealed a significant downregulation of CYP1A1 at the 1× and 10× exposure thresholds, potentially compromising the organisms’ cellular detoxification capacity, alongside a deregulation of pathways governing lipid metabolism and glucocorticoid receptor activities. This molecular response correlates directly with tissue concentrations, a trend also observed in other odontocetes, in which high POP burdens alter cellular homeostatic systems (Foltz et al. 2014; Panti et al. 2022). Furthermore, endocrine disruption can be severe, as noted in Florida dolphins, which showed high DDT levels associated with reduced testosterone in males and suppressed cortisol in females, thereby impairing reproduction and stress responses (Galligan et al. 2019). Finally, emerging evidence suggests that such high pollutant burdens can significantly alter the skin microbiome of endangered populations, further compromise immunity, and increase disease susceptibility (Jia et al. 2022).

The pervasive contamination of marine mammals underscores the failure of global policies to mitigate legacy pollutants, as known toxicity thresholds were frequently exceeded by 65.2% (15/23 studies) of the articles reviewed herein. Concentrations exceeding 9000 ng/g l.w. have been established as a critical threshold for immunosuppression in aquatic mammals, based on evidence that PCB concentrations above this level significantly increase the risk of infectious disease mortality by reducing immune competence (Kannan et al. 2000; Jepson et al. 2005). This limit was surpassed in several populations discussed in this review, independent of the region. This includes apex predators in the Mediterranean, such as long-finned pilot whales (*Globicephala melas*) (Pinzone et al. 2015) and Cuvier’s beaked whales (*Ziphius cavirostris*) (Baini et al. 2020), as well as coastal populations of bottlenose dolphins (*Tursiops truncatus*) in the Atlantic (García-Alvarez et al. 2014; Neely et al. 2018), the Gulf of Mexico (Balmer et al. 2019), and the Indian Ocean (Dirtu et al. 2016). We highlight that 80% of Cuvier’s beaked whales (*Ziphius cavirostris*) present contamination of PCBs 9000 ng/g (Baini et al. 2020).

Immunosuppression, infertility, transgenerational contamination, and neonatal mortality threaten the recovery of endangered species such as equatorial humpback stocks (Remili et al. 2020). In contrast with cetaceans, pinniped populations showed lower vulnerability depending on their foraging grounds. Northern elephant seals (*Mirounga angustirostris*) from Guadalupe Island had PCB concentrations significantly below toxicological concern, with a median of 331,4 ng/g d.w. (Muntaner-Lopez et al. 2023). This divergence emphasizes that, while pinnipeds are essential sentinels, their risk levels are highly habitat-dependent, as also observed in coastal versus oceanic dolphin populations (Dirtu et al. 2016). Furthermore, the alarming threshold 17,000 ng/g l.w. for reproductive impairment in cetaceans (Jepson et al. 2005) was also exceeded in several populations, including Adriatic dolphins (Genov et al. 2019) and tropical odontocetes, such as false killer whales (*Pseudorca crassidens*) from Hawaii (Foltz et al. 2014). These findings underscore the urgent need for global action to mitigate the impacts of pollution on marine mammals and human health, particularly among Arctic communities that consume cetacean blubber (Desforges et al. 2024). It is important to note, however, that even as global concentrations decline, evidence suggests that PCB-associated toxic effects and infectious disease risks may persist even in individuals slightly below these traditional thresholds, indicating that current safety limits might still underestimate the chronic pressure on endangered populations (Williams et al. 2020).

### Inorganic contaminants—potentially toxic metals

In studies on inorganic contaminants (Table 2), specifically metal and metalloid, eight scientific articles were identified, spanning the years 2014 (*n* = 2), 2015 (*n* = 1), 2019 (*n *= 2), 2020 (*n* = 2), and 2023 (*n* = 1). The metals analyzed included mercury (Hg; *n* = 6), arsenic (As; *n* = 3), lead (Pb; *n* = 2), chromium (Cr; *n* = 2), selenium (Se; *n* = 2) nickel (Ni; *n* = 1), iron (Fe; *n* = 1), magnesium (Mg; *n* = 1), zinc (Zn; *n* = 1), aluminum (Al; *n* = 1), beryllium (Be; *n* = 1), uranium (U; *n* = 1), cadmium (Cd; *n* = 1), antimony (Sb; *n* = 1), gold (Au; *n* = 1), barium (Ba; *n* = 1), tin (Sn; *n* = 1), lithium (Li; *n* = 1), cobalt (Co; *n* = 1), vanadium (V; *n* = 1), silver (Ag; *n* = 1), molybdenum (Mo; *n* = 1), strontium (Sr; *n* = 1), copper (Cu; *n* = 1), manganese (Mn; *n* = 1), and titanium (Ti; *n* = 1). It was found that two studies assessed more than one inorganic contaminant and, therefore, the frequency count exceeds the number of selected studies herein.

A series of analytical techniques has been used for determining inorganic contaminants in biosamples, including atomic absorption spectrometry (AAS; *n* = 2), notably utilizing Graphite Furnace detector (GF-AAS) for its high sensitivity in trace analysis (Pinzone et al. 2019; Barragán-Barrera et al. 2023); inductively coupled plasma mass spectrometry (ICP-MS; *n* = 2), prioritized for multi-elemental determinations (Savery et al. 2014; Wise et al. 2019). Other techniques included inductively coupled plasma atomic emission spectrometry (ICP-AES; *n* = 1) (Monk et al. 2014) and direct mercury analysis (DMA; *n* = 2), the latter providing a specialized approach for Hg without the need for complex acid digestion (Lian et al. 2020; Alava et al. 2020). Additionally, two studies use a combination of those techniques, i.e., atomic absorption spectrometry with direct mercury analysis (AAS and DMA; *n* = 1) and inductively coupled plasma mass spectrometry with inductively coupled plasma optical emission spectrometry (ICP-MS and ICP-OES; *n* = 1). The ICP-MS technique was prioritized across these studies for determining the inorganic elements due to its superior sensitivity and lower detection limits, whereas ICP-AES and ICP-OES were effectively applied to quantify abundant macroelements (Monk et al. 2014; Wise et al. 2019). Geographically, the studies included in this section, such as those in the POPs’ section, exhibit concentration in the Northern Hemisphere, particularly in the Gulf of Maine in the Northwest Atlantic (Wise et al 2015, 2019), the California coast (Lian et al. 2020) and the Mediterranean Sea (Pinzone et al 2019; Savery et al 2014). In contrast, data from the Southern Hemisphere remain sparse, comprising localized along the Pacific coast of South America (Alava et al. 2020), southeastern Australia (Monk et al. 2014), and remote polar Antarctic Peninsula (Barragán-Barrera et al. 2023). This spatial discrepancy underscores a historical monitoring bias toward industrialized northern coastlines.

Inorganic contaminants, such as metals, occur naturally but are amplified by anthropogenic activities (e.g., industrial emissions, agriculture). The toxic potential of a metal is not a fixed property, as it depends on several factors, including its chemical form, dose, route, and duration of exposure, as well as the biological context (Egorova and Ananikov 2017). While elemental forms are often low in toxicity, cationic species bind organic molecules, enabling bioaccumulation and biomagnification (Islam and Tanaka 2004; Zeng et al. 2015). Biopsies, despite limitations such as tissue heterogeneity and lower historical baselines in calves that directly reflect reduced accumulation and exposure time in early life stages (Lian et al. 2020), remain vital non-invasive tools for assessing contamination in threatened marine mammals.

The analysis of studies evaluating inorganic contaminants primarily highlights that, in contrast to POPs, the primary target tissue (or biopsy matrix) for analysis was the skin (epidermis), a preference likely attributed to the non-lipophilic nature of these inorganic pollutants. Unlike POPs, metals exhibit a high affinity for proteins, particularly those rich in sulfhydryl groups such as keratin (Kakuschke et al. 2005). Consequently, the epidermis functions as a metabolically active tissue rich in keratin and binding proteins and serves as a primary site of accumulation for toxic elements, providing a reliable proxy for internal organ burdens (Savery et al. 2014; Wise et al. 2019). While blubber remains the standard for lipid-soluble compounds, such as POPs and other lipophilic compounds, the protein-rich nature of the skin makes it a superior target matrix for capturing the long-term accumulation of non-lipophilic inorganic contaminants (Aubail et al. 2013; Andvik et al. 2023). This requirement for matrix optimization is further supported by internal epidermal variations, in which specific structural and hydration differences across strata necessitate precise sampling of active layers (Lian et al. 2020). The toxicological footprint of mercury (Hg) across different oceans reveals how skin biopsies function as an effective mirror of ocean health, registering distinct ecological and regional pressures. The Hg was predominant and present in almost all studies (6/8 articles), probably due to its persistence, biomagnification, and neurotoxic effects (McDermott et al. 2015; Behrens et al. 2023).

The interspecific heterogeneity in metal concentrations observed reflects trophic strategies and prey diversity, as shown by Pinzone et al. (2019). In the Mediterranean Sea, pilot whales (*Globicephala melas*) exhibited the highest Hg concentrations (33,028 ng/g) and the widest contamination niche due to their diverse diet, compared to specialized sperm whales (*Physeter macrocephalus*; 10,763 ng/g) and fin whales (*Balaenoptera physalus*; 814 ng/g). In fact, apex predators like sperm whales accumulate substantial Hg via high-trophic prey (e.g., mesopelagic squid) (Pinzone et al. 2019). While nitrogen stable isotopes (δ^15^N) validate the high trophic position of these apex odontocetes, global analyses report that the closed, oligotrophic nature of the Mediterranean basin acts as an environmental amplifier, driving Hg biomagnification through elongated pelagic food webs far more intensely than in an entirely open ocean system (Martínez-López et al. 2019; Delgado-Suarez et al. 2023).

This geographical and habitat-driven divergence is sharply evident when contrasting semi-enclosed seas with open pelagic ecosystems. For instance, trace element screenings conducted on stranded odontocetes in the Canary Islands Atlantic waters reveal significantly lower baseline Hg contents, yet highlight how deep-diving species, such as *Physeter macrocephalus* and *Globicephala macrorhynchus*, are uniquely exposed to distinct metal circulation pathways via the ingestion of high-trophic mesopelagic cephalopods, which act as primary vectors for transferring sediment-associated contaminants like cadmium (Cd) to top predators (Lozano-Bilbao et al. 2021; Delgado-Suarez et al. 2023). Conversely, coastal ecotypes of *Tursiops truncatus* foraging in shallower waters tend to accumulate elevated contents of anthropogenically mobilized elements like lead (Pb), chromium (Cr), and nickel (Ni) due to their proximity to urban and industrial runoff zones (Lozano-Bilbao et al. 2021). Ultimately, these multi-ocean comparisons demonstrate that interspecific contamination patterns cannot be evaluated solely through trophic levels; rather, they are modulated by a synergistic mix of species-specific feeding preferences, transboundary atmospheric transport, and localized coastal degradation (Lozano-Bilbao et al. 2021; Delgado-Suarez et al. 2023).

Inorganic contamination by potentially toxic metals, as Hg, also presents a geographic vulnerability. The resident bottlenose dolphins (*Tursiops truncatus*) from the Pacific coast of South America (Gulf of Guayaquil) showed alarming Hg concentrations (Table 2), with all sampled individuals exceeding immunotoxic thresholds, highlighting the severe pressure faced by populations near industrialized coastal zones (Alava et al. 2020). Two of the studies demonstrate evidence of contamination by different metals exceeding the limits considered safe for marine mammals. Regarding thresholds, according to a review on neurotoxic effects of Hg in marine mammals by López-Berenguer et al. (2020), understanding the true extent of this contamination's damage requires comparison with established systemic limits.

In marine mammals, Hg accumulation in the skin indicates that internal storage capacity has been exceeded, signaling that this capacity approaches dangerous toxicological levels. Assessments in other Arctic marine mammals indicate that physiological disruption and hepatic risk are expected when internal thresholds exceed approximately 200,000 ng/g of wet weight (Dietz et al. 2022), as observed in many of the marine mammals in this review (Table 2). Furthermore, a data compilation by Kershaw and Hall (2019) shows that free-ranging cetaceans expressed hepatotoxicity and neurotoxicity effects emerging from 60,000 ng/g d.w. and 400 ng/g d.w., respectively, as also observed in cetaceans that are included in the present review.

Intraspecific differences, such as 37% higher Hg in females of Southern elephant seals (*Mirounga leonina*) compared to subadults (Barragán-Barrera et al. 2023), arise from mesopelagic foraging and maternal transfer. Habitat and urbanization impacts are also a relevant factor; Monk et al. (2014) confirmed that stranded Burrunan dolphins (*Tursiops australis*) from industrialized regions in Australia accumulated blubber Hg levels (mean: 3640 ng/g) nearly three times higher than live individuals (mean: 1320 ng/g). This disparity provides direct evidence of chronic dietary bioaccumulation, which was supported by the simultaneous detection of measurable Hg in their prey fish, confirming food webs as the primary exposure vector (Sun et al. 2020). Furthermore, this chronic contamination is evidenced by the massive accumulation observed in internal organs of the necropsied individuals, with hepatic concentrations reaching critical levels up to 840,000 ng/g (Monk et al. 2014). This systematic overflow from hepatic storage to peripheral tissues over time demonstrates that long-term dietary ingestion outpaces natural detoxification and elimination rates throughout the individual lifespan (Kershaw and Hall 2019). Similarly, Monk et al. (2014) reported Hg concentrations exceeding immunotoxic thresholds in dolphins (Alava et al. 2020: 1920–3630 ng/g) highlighting risks in industrialized coastal regions. Furthermore, the observed formation of tiemannite (i.e., Hg: Se ≈1: 1.06—Monk et al. 2014) indicates active detoxification but signals of chronic physiological stress, as Hg-Se binding depletes Se reserves essential for vital antioxidant and immunoprotective functions in animals (Faganeli et al. 2018).

Beyond Hg, skin biopsies have expanded our understanding of global baseline exposures to other highly hazardous inorganic elements, such as arsenic and chromium, which are poorly monitored in live populations (Sun et al. 2020). Savery et al. (2014) established the first global baseline for As in free-ranging sperm whales across 17 oceanic regions, detecting the element in 98.5% of samples (global mean of 1900 ng/g). It is important to emphasize that these results represent the environmental conditions of the 2000–2005 sampling period. Establishing such historical benchmarks is essential for long-term ecotoxicological monitoring, as it allows for a retrospective comparison with more recent data. The highest accumulations occurred in the Indian Ocean (2100 ng/g, peaking at 15,600 ng/g in the Seychelles) and the Mediterranean Sea, driven by a squid-based diet and regional anthropogenic discharges, with males accumulating higher levels than females and evidence of partial excretion in shed skin samples.

Contaminants such as Cr and Ni (Wise et al. 2019) raise concerns due to their carcinogenic and reproductive effects (Behrens et al. 2023). The in vitro toxicity of Cr(VI) in fin whale skin cells exposed to sodium or lead chromates demonstrated dose-dependent cytotoxicity and genotoxicity, suggesting long-term population impacts, including reduced cell viability and disease susceptibility (Wise et al. 2015). In turn, Se, quantified in two studies, showed 3.27-fold higher concentrations in whales than dolphins (Monk et al. 2014; Wise et al. 2019), consistent with the higher trophic position of the former marine mammals (Pinzone et al. 2019). While Se has immunoprotective roles (Schrauzer 2009), its Hg-binding (Hg:Se formation) reduces bioavailability, risking DNA damage (Letavayová et al. 2006), and compromising antioxidant functions. Thus, despite higher whale concentrations reflecting expected bioaccumulation, Se–Hg synergy necessitates continuous monitoring in polluted ecosystems to assess long-term health impacts and the escalating toxicological degradation of the global marine environment.

### Emerging pollutants—plasticizers

Regarding emerging contaminants (Table 3), this review included two scientific articles, published in 2016 (*n* = 1) and 2024 (*n* = 1). A total of eight contaminants were analyzed by GC-MS (Sambolino et al. 2024) and GC-ECD (Fossi et al. 2016), including seven phthalate esters: di-(2-ethylhexyl) phthalate (DEHP), dimethyl phthalate (DMP), diethyl phthalate (DEP), di-isobutyl phthalate (DIBP), di-*n*-butyl phthalate (DBP), benzyl-butyl phthalate (BBP), and di-*n*-octyl phthalate (DNOP), and MEHP (mono-(2-ethylhexyl) phthalate), a metabolite of DEHP. These screening results highlight the viability of remote biopsies to track modern plastic pollution, the “invisible” plastic pollution in free-ranging marine mammals, shifting the focus from historical legacy pollutants to escalating contemporary threats impacting the marine environment.

The evaluation of emerging pollutants via biopsy sampling is uniquely represented in this review by studies targeting plastic additives, specifically phthalate acid esters (PAEs) and their key metabolic breakdown products. Methodologically, the articles focused exclusively on gas chromatography coupled with mass spectrometry (GC-MS) to identify and quantify these lipophilic plasticizers from multi-species biopsy matrices. Geographically, the monitoring is entirely localized within the Northern Hemisphere, specifically in the semi-enclosed basin of the Mediterranean Sea, Pelagos Sanctuary, and the Northeast Pacific coast along the Gulf of California, both sampling areas of Fossi et al. (2016) and the Northeast Atlantic waters surrounding the Madeira Archipelago in Sambolino et al. (2024).

Emerging pollutants are anthropogenic chemical substances or biological agents (e.g., pharmaceuticals, personal care products, plasticizers, and nanomaterials) that, despite decades of environmental presence, have only recently been identified as potential threats to human and ecosystem health (Sarma 2022). Their detection is often limited by insufficient regulation, analytical challenges, or underestimation of their effects at low concentrations (Bell et al. 2011; Tang et al. 2019). Examples include pharmaceuticals, personal care products, plasticizers, and nanomaterials, which induce adverse effects such as oxidative stress, reproductive impairments, carcinogenesis, apoptosis, and endocrine and nervous system disorders (Grobelak and Kowalska 2022). Due to potential health effects, studies assessing pollutants such as phthalate esters have increased markedly. These contaminants have been widely quantified across various marine taxa, with marine mammals, such as cetaceans, being top predators susceptible to their metabolic impacts (Lima et al. 2025). Biopsy sampling, though underexplored for phthalate assessment, proved viable for monitoring contamination and evaluating ecotoxicological risks in marine mammals. Although Fossi et al. (2016) assessed microplastics, their analyses were based on water and zooplankton sampling rather than biopsies. Thus, only the results related to the fin whale’s (*Balaenoptera physalus*) phthalate contents are shown and discussed here.

Heterogeneity in phthalate exposure was evident in a species between different regions. Mediterranean fin whales (*Balaenoptera physalus*) (Fossi et al. 2016) exhibited 35% higher MEHP concentrations (mean: 54.8 ng/g) than those in the Gulf of California (40.0 ng/g), with seasonal peaks in the Ligurian Sea reaching up to 65.5 ng/g. This 35% higher concentration reflects greater anthropogenic pressure in that semi-enclosed sea, where coastal activities (tourism and plastic industries) and heavy shipping routes likely contribute to the release of plasticizers. Additionally, marine debris fragmentation and degradation of marine debris release substantial phthalates into the Mediterranean Sea (Suaria and Aliani 2014; Cózar et al. 2015; Paluselli et al. 2019).

In the NE Atlantic, specific ecological interactions and trophic niches also mediate the contamination of emerging pollutants. Common bottlenose dolphins (*Tursiops truncatus*) from the Madeira Archipelago showed a fourfold higher total phthalate burden (ΣPAEs = 947.56 ng/g) compared to sympatric short-finned pilot whales (*Globicephala macrorhynchus*; ΣPAEs = 229.98 ng/g). Sambolino et al. (2024) suggest an association with the trophic niche and exposure. Trophic segregation in fatty acid profiles underscores the connection between dietary habits, metabolism, and contamination patterns. Pilot whales (*Globicephala* sp.), for example, feeding on mesopelagic squid, may ingest phthalates associated with plastic debris in deep-water zones, where microplastics act as transport vectors (Sambolino et al. 2023). Conversely, dolphins, consuming epipelagic fish rich in DHA and EPA, are more exposed to DEHP—a plasticizer widely used in food packaging and hygiene products that enters the food chain via coastal runoff (Jambeck et al. 2015). In these dolphins, DEHP was the major ester detected at 100% frequency (mean of 778.12 ng/g) (Sambolino et al. 2024).

These findings corroborate patterns observed in marine mammals, where DEHP and its metabolites are detected at high concentrations (Routti et al. 2021; Page-Karjian et al. 2020; Dziobak et al. 2022). The detection of DEHP in all dolphin samples (Sambolino et al. 2024) was also observed in a coastal cetacean, the Franciscana dolphin, also known as Toninha (*Pontoporia blainvillei*), in the South Atlantic Ocean (Lima et al. 2024a) and in 92,8% of occurrences in a river species, i.e., the Amazon River Dolphin (*Inia geoffrensis*) (Lima et al. 2024b). This is alarming, as DEHP acts as an endocrine disruptor, impairing fertility, embryonic development, and hepatic function in mammals (Hannon and Flaws 2015; Zhan et al. 2022), besides causing cellular damage (Hlisníková et al. 2020). Importantly, the extreme DEHP outlier (4697.34 ng/g) found in a bottlenose dolphin underscores the need for continuous monitoring to identify contamination hotspots (Sambolino et al. 2024). Similarly, DBP prevalence (80% in pilot whales) warrants attention given its associations with oxidative stress, hormonal disruption, and reproductive complications in mammals (Aly et al. 2016). Although no direct correlation between PAEs and lipid alterations was found in the short term, chronic exposure to phthalates may disrupt lipid metabolism over the long term, warranting ongoing monitoring in marine ecosystems.

## Gaps and perspectives

This systematic review focused on the period 2014–2024 and identified that biopsy sampling in marine mammals has become an indispensable tool for studying contamination by various pollutants in recent years. Unlike stranded animals, which rely on opportunistic sampling (often representing debilitated or deceased individuals), biopsies provide direct access to contaminant data from free-ranging, healthy populations, offering a real-time snapshot of ecosystem health. This technique not only circumvents the biases inherent in stranding-based studies (e.g., underrepresentation of robust populations or specific age classes) but also enables longitudinal monitoring of contamination trends in dynamic marine environments. For instance, while stranded specimens may reflect acute or end-stage exposure, biopsies reveal baseline pollutant levels in active populations, critical for assessing acute and chronic impacts and formulating preemptive conservation strategies (Fossi et al. 2016; Desforges et al. 2024). However, despite its relevance, notable gaps were identified in the reviewed literature, limiting its full potential and suggesting pathways for future advancements.

POPs, such as PCBs and DDTs, dominated the studies, followed by metals and emerging pollutants like phthalates. Phthalates, a class of emerging contaminants, are severely understudied. Only two studies (Fossi et al. 2016; Sambolino et al. 2024) detected phthalates in marine mammals via biopsy sampling, with DEHP concentrations reaching 4697 ng/g in bottlenose dolphins. Given the widespread contamination of marine organisms with phthalates, more studies are crucial to identifying their presence and metabolic interference in marine mammals (Zhang et al. 2021). This is particularly alarming given that the widespread ingestion of plastic debris is a primary physical vector for plasticizers, creating a chronic sublethal pathway for chemical exposure among coastal populations (Valente et al. 2026). Additionally, the absence of toxicity thresholds for these endocrine disruptors impedes risk assessment, particularly in tropical regions where intensive plastic use and the thermal degradation of these polymers accelerate phthalate release (Paluselli et al. 2019). The lack of studies on other emerging pollutant classes, such as pharmaceuticals, per- and polyfluoroalkyl substances (PFAS), and other plasticizers like bisphenol A (BPA), underscores the underutilization of biosamples as indicators of marine pollution.

Regarding the synergistic interactions among the different contaminants, only two studies assessed the presence of pollutants from the different categories reviewed herein but did not evaluate combined toxicity effects. The presence of multiple pollutants could amplify toxicity and modify metabolic processes, leading to population declines in already vulnerable species (Cabral et al. 2019; Fleury and Baulin 2024).

Cetaceans, especially bottlenose dolphins (*Tursiops truncatus*), dominate research while pinnipeds are underrepresented. This result is expected, as biopsy sampling in pinnipeds often requires capturing live individuals, and holding and sedating them, making the procedure quite stressful and invasive (Steinmetz et al. 2021). Although pinniped biopsy procedures remain challenging, their implementation can be a valuable tool, as they reveal important information about the group’s risks of pollutant contamination. More detailed investigations into how this group responds to pollutants become highly relevant. Pinnipeds are not only bioindicators of pollution but also umbrella species that highlight the distribution of pathogens, thereby reflecting the real status of the marine environment and public health (Barratclough et al. 2023; Lima et al. 2024c). Another key group of marine mammals that inhabits only the Southern Hemisphere, including the Southern right whale (*Eubalaena australis*), the Amazonian manatee (*Trichechus inunguis*), and otters, remains neglected in studies assessing contamination in free-ranging animals. Another gap is the scarcity of studies evaluating the direct relationship between contamination in marine mammals and their prey. Only Monk et al. (2014) simultaneously analyzed mercury in dolphins and prey fish, revealing biomagnification. The absence of such data hinders understanding of trophic mechanisms, such as key prey vectors for contaminants or how seasonal dietary shifts modulate exposure.

A significant gap observed in this systematic review is the geographical bias. The predominance of studies in North America and Europe leaves relevant regions like Central and South Americas, Australia, and Asia understudied. It is well-known that such continents experienced intense anthropogenic activities that cause, for instance, improper plastic disposal and chemical contamination of marine ecosystems (Jambeck et al. 2015; Jang et al. 2017; Aranda et al. 2022; Alencar et al. 2023). In Australia, only two studies used biopsy sampling to investigate contamination in cetaceans. As reviewed by Jarolimek et al. (2023), most inorganic contaminant data in Australian marine fauna relies on opportunistic sampling from stranded carcasses or bycatch. This dependence introduces geographic and temporal biases, while the lack of baseline data from healthy, uncontaminated individuals limits accurate toxicological interpretations. Consequently, the scarcity of studies using non-invasive remote biopsy sampling leaves a diagnostic blind spot for monitoring the real-time health status of free-ranging and vulnerable coastal populations (Monk et al. 2014; Jarolimek et al. 2023). Moreover, ecosystems such as Brazil’s “Blue Amazon” (marine territory) and Uruguayan/Argentine coast (i.e., Mar del Plata), where intense pollution releases contaminants like toxic metals, POPs, and plastics, require urgent efforts for marine mammal conservation (Villagran et al. 2021; Montone et al. 2023; Belli et al. 2024). This urgency is critically amplified by recent legislative shifts, such as the new Brazilian Pesticide Law (Lei n° 14.785/2023, known as “PL do Veneno”), which replaces stringent “danger” criteria with flexible “risk” assessments for new agrochemicals (BRASIL 2023). The documented enduring toxic legacy of banned POPs, such as DDTs and PCBs, in marine mammals globally, coupled with the acute lack of contamination baseline data for South America, underscores a potential catastrophic regulatory failure: a new wave of contaminants could be authorized for use in these ecologically sensitive, yet scientifically under-monitored, regions.

The geographical disparity could be attributed to constant insufficient financial and technological investment in science in Latin America. Despite the growth in scientific productivity in marine science in Brazil (Potter and Pearson 2023), America Latina, as a whole, still has drastic budget cuts in science and technology, leading to the disruption of research and social inequality that directly impacts education in these countries (Humberto 2025). The solution lies in political coordination, global cooperation, and strategic investment in science in this region. In global collaboration, partnerships between public and/or private organizations could provide alternatives, such as technical support and logistics for monitoring the health of free-ranging marine mammals. Another alternative is the expansion of international data banks, such as the National Marine Mammal Tissue Bank (NOAA 2023), to include free-ranging marine mammal samples.

The lack of standardization in collection, storage, and analytical protocols also emerges as a significant barrier. Studies such as Genov et al. (2019) and Balmer et al. (2019) employed different chromatographic methods (GC-ECD vs. GC-MS) to quantify POPs, resulting in differences in PCB-153 concentrations of up to 40% (a key congener for risk assessment). Additionally, discrepancies in POPs detection limits and compound recovery rates hinder cross-study comparisons. Integrating non-destructive approaches, such as Raman spectroscopy, could address these gaps, since its ability to identify polymers (e.g., micro- and nanoplastics; Erni-Cassola et al. 2017) and correlate chemical contamination with tissue metabolic changes offers a complementary layer to traditional GC-MS or LC-MS workflows. Moreover, to address this limitation, we propose adopting standards based on high-resolution techniques (e.g., GC-HRMS), establishing reference certificates for marine mammal tissues and following successful models, such as those of the National Institute of Standards and Technology (NIST). The absence of standardized guidelines for tissue parameters (e.g., biopsy fat thickness, shown to be relevant in Lian et al.’s study) and storage conditions (which varied widely among studies kept on ice, –20 °C, –80 °C, and liquid N_2_) may introduce biases, particularly in studies focusing on metabolic analysis (Sambolino et al. 2024). The lack of standardization in collection, storage, and analysis protocols can severely compromise conservation efforts. Best practices in sampling and preservation enhance data quality and enable collaborative, multi-institutional research efforts (Van Cise et al. 2024). Non-comparable data hinder accurate assessments of the impacts of contamination on marine mammal populations, leading to inadequate conservation decisions. This may result in underestimating or overestimating the risks faced by vulnerable species, directly affecting the effectiveness of protection strategies. Standardization is essential to ensure that the collected data is reliable and useful for formulating effective conservation policies (Sequeira et al. 2021).

The scarcity of studies integrating biopsy sampling with long-term monitoring is a significant obstacle. While snapshot studies, such as Baugh et al. (2023) on humpback whales (*Megaptera novaeangliae*) in the Gulf of Maine, revealed contamination peaks in juveniles (up to 12,000 ng/g PCBs), the absence of continuous monitoring hinders understanding of chronic effects like immune dysfunction or reduced fertility across life cycles. Although Genov et al. (2019) and Hayes et al. (2022) conducted multi-year sampling (2003–2017 and > 5 years, respectively), irregular sampling frequencies and limited sample sizes (*n* = 32 in Genov et al. 2019) prevent robust population-level conclusions. Continuous biopsy-based monitoring is highly effective for identifying genetic patterns, habitat use, and trophic ecology (Fruet et al. 2015; Genoves et al. 2020; Troina et al. 2020), offering significant potential to advance knowledge on marine mammal contamination. Studies such as Desforges et al. (2024) on Arctic killer whales (*Orcinus orca*) highlight the importance of temporal data for predicting human health risks, particularly among certain native communities that consume cetacean blubber. However, such approaches remain exceptions.

Another important factor is the limited integration of biopsies with complementary techniques. The combination of ecological data, such as stable isotope (δ^13^C, δ^15^N) and contamination analyses, was partially explored in the reviewed studies and proved successful, but its application remains sporadic. Incorporating predictive modeling approaches could quantify the contribution of specific prey to pollutant bioaccumulation, as demonstrated in Baugh et al. (2023) for PCBs in humpback whales. These combined strategies would position stable isotopes as central tools for unraveling not only contamination levels, but also the ecological and anthropogenic dynamics that modulate them, enhancing biopsies’ potential as a holistic diagnostic tool for marine health. Studies like Tartu et al. (2020), which linked δ^15^N to mercury levels in blue whales, illustrate how diet and movement influence exposure. Techniques such as satellite telemetry track marine mammal movements, revealing habitat use patterns that better elucidate pollutant exposure and the role of contamination hotspots in mobile predators (Brown et al. 2014; Laidre et al. 2022). In a complementary approach, machine learning platforms can be used to predict contamination hotspots, such as algorithms that identify marine plastic debris (Jalil et al. 2023). This could involve integrating biopsy data and oceanographic variables to identify marine areas in need of protection.

Furthermore, integrating multi-omics approaches (genomic, proteomic, transcriptomic, metabolomic, and lipidomic) with population data could identify genetically resilient individuals or populations, advancing conservation efforts (Van Cise et al. 2024). Omics methods can play a crucial role in contamination detection and marine conservation by providing advanced methodologies for monitoring biodiversity and assessing pollutant impacts. Omics technologies have elucidated physiological responses in marine mammals, such as dolphins, to environmental stressors, aiding ecotoxicology studies (Collí-Dulá and Ruiz-Hernández 2022; Lima et al. 2024a, b, c) and could contribute to managing Marine Protected Areas (MPAs) and enhancing conservation strategies for threatened species.

Finally, filling these gaps is a fundamental requirement to advance the One Health framework worldwide. The One Health approach recognizes that human, animal, and environmental health are deeply interconnected and has as principles equity, sociopolitical and multicultural parity, socioecological equilibrium, stewardship in companies, transdisciplinarity, and multisectoral collaboration (Adisasmito et al. 2022; Norman et al. 2023). In this context, the toxicological profiles compiled in this review emphasize the role of marine mammals as ecological sentinels, a role recognized for decades (Bossart 2011), particularly in linking ocean degradation to human well-being. Cetaceans and Pinnipeds share high trophic positions and long lifespans with human coastal populations, and the alarming burdens of persistent organic pollutants (POPs) and mercury (Hg) detected via non-invasive biopsies serve as a direct proxy for shared dietary and environmental risks. This toxicological connection is evident when evaluating traditional and local communities that historically rely on marine resources for food security. In these populations, the chronic ingestion of contaminated seafood poses severe public health threats, including neurodevelopmental delays, immunotoxicity, and endocrine disruption (Desforges et al. 2024). Indeed, the simultaneous detection of inorganic contaminants such as mercury in dolphins and their prey (Monk et al. 2014) highlights contamination of shared food webs that affects human food security. Since this shared seafood constitutes a primary source for human populations, continuous monitoring of pollutant accumulation becomes a public health priority (Massone et al 2023; Garofalo et al. 2025).

In ports and coastal areas of South America, edible fish, mollusks, and invertebrates have been observed to accumulate mixtures of prohibited contaminants chronically (Primost et al. 2024; Ferreira et al. 2026). Dietary exposure models for these southern communities reveal that shared consumption can exceed intake limits considered noncarcinogenic (Rodríguez-Hernández et al. 2017; Ferreira et al. 2026). Therefore, the environmental persistence of these contaminants requires immediate intervention within the One Health framework. Furthermore, strengthening conservation policies, guided by continuous monitoring of marine mammals using non-invasive techniques such as remote biopsy, is an indispensable prerequisite for preventing public health crises and protecting vulnerable marine biodiversity. In a climate change scenario, rising temperatures may alter the presence of pollutants in the environment, resulting in redistribution, transformation, and remobilization of old contamination, shifting it between environmental compartments (Wang et al. 2016; Borgå et al. 2022; Hung et al. 2022). Consequently, these climate-driven pollutant dynamics highlight the urgency of long-term surveillance, where real-time toxicological analyses derived from non-invasive biopsies become paramount to forecast cascading chemical risks within shared trophic webs. Ultimately, mitigating the geographic and funding disparities identified here is imperative to eliminate existing “diagnostic blind spots” in global ocean health monitoring. Investment in science and international cooperation represents the only viable pathways to safeguard marine ecosystems and human communities within the One Health framework.

## Conclusion

The literature compiled over the last decade in this systematic review highlights the chronological, methodological, and taxonomic applications of biopsies as an efficient sampling method in marine mammal ecotoxicology. Notably, the sampling periods revealed that this review provides a retrospective assessment of ocean health spanning over two decades (2000–2024). Recognizing this temporal gap between data collection and publication is essential for accurately interpreting trends in legacy and emerging contaminants. The assessment of 31 studies on contamination by POPs, inorganic contaminants (potentially toxic metals), and emerging pollutants (plasticizers such as phthalates) revealed that POPs were the most investigated contaminants. Despite the detection of one plasticizer type (DEHP) in all samples from the three cetacean species analyzed, emerging pollutants such as phthalates have been rarely studied. The articles on PCBs and DDTs suggest that these persistent and historical pollutants pose a significant threat to marine mammals. The information on historical POPs contamination also alerts to the need for greater attention to phthalates in marine research, which have more than enough potential to become historical pollutants themselves.

Regarding the animals studied, cetaceans, particularly bottlenose dolphins (*Tursiops truncatus*), were the most analyzed species, while pinnipeds were less investigated. These results highlight the importance of focusing attention on species that share the marine ecosystem with cetaceans and that also face marine pollution and other environmental pressures, such as climate change.

Overall, minimally invasive sampling methodology presents a promising path to a better understanding of the current state of pollution in the marine ecosystem. This approach can be a powerful tool for the development of management policies aligned with the One Health vision. Moreover, the implementation of an integrated approach that combines longitudinal studies tracking trends in sentinel species with “omics” science can complement biopsy sampling. This combination enables the evaluation of the impacts of policies, such as POPs reductions driven by the Stockholm Convention, and promotes biodiversity conservation.

## Data Availability

All data and materials are available upon reasonable request to the corresponding author.

## Abbreviations

AAS: Atomic absorption spectrometry
BPA: Bisphenol A
BBP: Benzyl butyl phthalate
CHLs/∑CLHs: Chlordanes/total chlordanes
CYP1A1: Cytochrome P450 1A1
DBP: Di-n-butyl phthalate/dibutyl phthalate
DDTs/∑DDTs: Dichlorodiphenyltrichloroethanes/total dichlorodiphenyltrichloroethanes
DEHP: Di-(2-ethylhexyl) phthalate
DEP: Diethyl phthalate
DIBP: Di-isobutyl phthalate
DMP: Dimethyl phthalate
DNOP: Di-n-octyl phthalate
DP: Dechlorane plus
d.w.: Dry weight
GC-ECD: Gas chromatography with electron capture detector
GC-HRMS: Gas chromatography-high resolution magnetic sector mass spectrometry
GC-LRMS: Gas chromatography-low resolution mass spectrometry
GC-MS: Gas chromatography-mass spectrometry
GC-MSD: Gas chromatography-mass selective detector
HCB: Hexachlorobenzene
HCHs/∑HCHs: Hexachlorocyclohexanes/total hexachlorocyclohexanes
HFRs/∑HFRs: Halogenated flame retardants/total halogenated flame retardants
ICP-AES: Inductively coupled plasma atomic emission spectrometry
ICP-MS: Inductively coupled plasma mass spectrometry
ICP-OES: Inductively coupled plasma optical emission
LOQ: Limit of quantification
l.w.: Lipid weight
M-PCBs: Mono-ortho polychlorinated biphenyls
MEHP: Mono-(2-ethylhexyl) phthalate
PAEs/∑PAEs: Phthalate esters/total phthalate esters
PAHs/∑PAHs: Polycyclic aromatic hydrocarbons/total polycyclic aromatic hydrocarbons
PBDEs/∑PBDEs: Polybrominated diphenyl ethers/total polybrominated diphenyl ethers
PCBs/∑PCBs: Polychlorinated biphenyls/total polychlorinated biphenyls
PCNs: Polychlorinated naphthalene
POPs/∑POPs: Persistent organic pollutants/total persistent organic pollutants
p,p′-DDE: Dichlorodiphenyldichloroethylene
PRISMA: Preferred Reporting Items for Systematic Reviews and Meta-Analyses
RNA seq: RNA sequencing
TRVs: Toxicity reference values
